# Structure and conductivity of ionomer in PEM fuel cell catalyst layers: a model-based analysis

**DOI:** 10.1038/s41598-023-40637-0

**Published:** 2023-08-29

**Authors:** W. Olbrich, T. Kadyk, U. Sauter, M. Eikerling, J. Gostick

**Affiliations:** 1 Theory and Computation of Energy Materials (IEK-13), Institute of Energy and Climate Research, Forschungszentrum Jülich GmbH, 52425 Jülich, Germany; 2 Robert Bosch GmbH, Corporate Research, 71272 Renningen, Germany; 3 Chair of Theory and Computation of Energy Materials, Faculty of Georesources and Materials Engineering, RWTH Aachen University, 52062 Aachen, Germany; 4 Jülich Aachen Research Alliance, JARA Energy, 52425 Jülich, Germany; 5 Department of Chemical Engineering, University of Waterloo, Waterloo, ON Canada

**Keywords:** Computational methods, Fuel cells

## Abstract

Efforts in design and optimization of catalyst layers for polymer electrolyte fuel cells hinge on mathematical models that link electrode composition and microstructure with effective physico-chemical properties. A pivotal property of these layers and the focus of this work is the proton conductivity, which is largely determined by the morphology of the ionomer. However, available relations between catalyst layer composition and proton conductivity are often adopted from general theories for random heterogeneous media and ignore specific features of the microstructure, e.g., agglomerates, film-like structures, or the hierarchical porous network. To establish a comprehensive understanding of the peculiar structure-property relations, we generated synthetic volumetric images of the catalyst layer microstructure. In a mesoscopic volume element, we modeled the electrolyte phase and calculated the proton conductivity using numerical tools. Varying the ionomer morphology in terms of ionomer film coverage and thickness revealed two limiting cases: the ionomer can either form a thin film with high coverage on the catalyst agglomerates; or the ionomer exists as voluminous chunks that connect across the inter-agglomerate space. Both cases were modeled analytically, adapting relations from percolation theory. Based on the simulated data, a novel relation is proposed, which links the catalyst layer microstructure to the proton conductivity over a wide range of morphologies. The presented analytical approach is a versatile tool for the interpretation of experimental trends and it provides valuable guidance for catalyst layer design. The proposed model was used to analyze the formation of the catalyst layer microstructure during the ink stage. A parameter study of the initial ionomer film thickness and the ionomer dispersion parameter revealed that the ionomer morphology should be tweaked towards well-defined films with high coverage of catalyst agglomerates. These implications match current efforts in the experimental literature and they may thus provide direction in electrode materials research for polymer electrolyte fuel cells.

## Introduction

Polymer electrolyte fuel cells (PEFCs) will be a key technology of a future sustainable energy ecosystem. At the brink of commercialization, further advances in performance and durability are needed^1^. A major proportion of irreversible performance losses originate in transport processes in the gas diffusion electrodes. This applies to all species involved in the overall fuel conversion, namely oxygen, hydrogen, protons, and electrons as well as water in liquid and vapor state. In earlier stages of fuel cell development, proton conductivity was not as critical as it is in today’s high-performing cells. The introduction of perfluorosulfonic acid (PFSA) polymers, such as Nafion, as highly charged electrolytes in the late 1980s^2,3^, alleviated the problem of proton supply in the cathode catalyst layer (CCL). However, during the last two decades large improvements of water management, oxygen supply and membrane resistance finally cleared the path to higher current densities and higher specific power output. With this progress, the proton conductivity in the catalyst layer emerged again as a crucial performance bottleneck^4^.

The ionomer content in the catalyst layer is a key optimization parameter: on the one hand, increasing the ionomer content increases the proton conductivity; on the other hand, excessive amounts of ionomer will block the pore space and thus impair gas transport^5^. Finding the optimal ionomer content has thus been the subject of several modelling studies^6–10^ and experimental efforts^11–13^. An optimal ionomer volume fraction of $\approx 30...40\%$ has been reported in the literature^7,8,10, 12–14^, though the exact value can vary significantly, with a range from 13 to $40\%$ found in commercially available catalyst layers^14,15^. These differences might have their origin in the varying microstructure of the CCL^16^. The optimal ionomer content also depends on operation conditions, catalyst layer thickness and Pt loading^13^. The ionomer content (in relation to Pt/C content) controls the interplay of transport (oxygen, water, and protons) and reaction, and thus can be tuned to optimize this interplay^6,10^. However, the relation between CCL microstructure and proton conductivity is not yet fully understood. Therefore, this work puts a primary focus on ionomer coverage and film thickness in the agglomerated CCL microstructure, which seem to be key parameters shaping proton conductivity. Molecular level structural features matter in this context, as water layer formation and structural ordering could both enhance or impair the proton mobility^17,18^

In conventional CCLs, Pt/C catalyst particles aggregate and are partially covered by a thin ionomer film with $\approx 5...15~\text{nm}$ thickness^19,20^. Closer examination of this agglomerate structure and of the ionomer morphology revealed that typically the ionomer film is unevenly distributed^21–24^, i.e., its coverage and thickness can vary significantly. Additionally, large ionomer aggregates that are not part of the thin film have been observed^24–26^. These variations in ionomer morphology largely impact the percolation behavior of the ionomer network and thus the proton conductivity of the CCL, as well as the volume fraction and network properties of gas-filled pores needed for oxygen supply^27–29^. The ionomer morphology in the CCL is not only determined by the composition of the ink, but also by Pt loading, type of carbon support, type of ionomer, ink solvent and processing parameters like processing times and thermal treatments^16,30,31^.

The understanding of how the ionomer morphology can be tuned and which structures yield an optimal performance remains a subject of ongoing experimental efforts^24,32–35^. At present, fuel cell developers have to optimize the CCL composition and fabrication process individually for every material combination and fuel cell application. Models that correlate the aforementioned variations in microstructure with transport properties and performance could provide much-needed guidance in this process^10,36^.

### Analytical relations between proton conductivity and CCL composition

Structure- and composition-dependent expressions for the proton conductivity of the CCL often employ semi-empirical power-law relations such as the Bruggeman relation or they are derived from percolation theory. The Bruggeman relation, proposed in 1935^37,38^, is widely adopted for transport properties in heterogeneous media and provides a satisfying fit to experimental data in specific applications, e.g., gas diffusion in porous media^39^, thermal conductivity of composite materials^40^, or magnetic permeability of ferromagnetic composites^41^. It expresses the relative reduction of a transport coefficient in the medium compared to the known bulk value through a simple power law. The semi-empirical relation proposed by Bruggeman implies an exponent of 1.5. This value was also adopted in modeling works for the proton conductivity in PEFC catalyst layers^8,9,42, 43^. Other works suggested values of 1.0^44^ or 2.0^45,46^, leaving the value up to debate and revealing the lack of generality of the approach.

The original approach taken by Bruggeman^37,38^ to derive a volume-averaged transport coefficient originally assumed a spherical geometry of primary particles forming the the conductive phase. Hashin and Shtrikman extended Bruggeman’s approach to spherical particles covered by a uniform, conductive film^47^. Das et al.^48^ applied the coated sphere model of Hashin and Shtrikman to PEFC catalyst layers by introducing a second coating, which represents the pore space between agglomerates, and introduced a dependence on the void fraction in the CL material. Since the approach of Das et al. describes the upper limit of proton conductivity, an additional empiric factor was introduced to account for a potentially lowered conductivity due to effects of ionomer geometry and agglomerate shape, i.e., the proposed correlation could not analytically resolve the dependence on ionomer morphology.

Percolation theory offers another approach to relate the proton conductivity with ionomer content^6,7^. The mathematical fundamentals of percolation theory were originally derived by Broadbent and Hammersley in 1957^49^ and form a sound statistical-physical basis to describe transport properties in heterogeneous media^50,51^. Above the percolation threshold, the dependence of proton conductivity on ionomer volume fraction is given by a power-law, with a critical power-law exponent that depends solely on the dimension of the percolating system ($\approx 2$ in the 3D case), whereas the percolation threshold depends on the the lattice structure or morphology of the continuous phase. In contrast to the Bruggeman relation, percolation theory provides a direct connection between relevant parameters, i.e., critical exponent and percolation threshold, and the microstructure of the material of interest. Eikerling et al.^6^ suggested to use an ionomer volume fraction of 0.1...0.2 for the percolation threshold of ionomer in catalyst layers, indicating a high level of connectedness in the ionomer network. Additionally, percolation theory enables the prediction of other structural features, such as the interfacial area of different phases or the relative utilization of a randomly connected phase^10^.

A different approach was taken by Liu et al.^52^ who derived a relation assuming spherical, cubically packed catalyst particles that are all fully covered by an ionomer film of uniform thickness. To fit the model prediction to their dataset, the authors introduced an adsorbed ionomer volume of I:C $= 0.3$ and a roughness factor (*rf*) of 1.6. The proposed relation describes the singular dataset considered in their work well, but was of limited applicability to other CCL material combinations. Additionally, the proton conductivity does not converge to the value of bulk ionomer upon increasing the ionomer content. However, as common in the literature, Liu et al.^52^ assumed the proton conductivity of the polymer electrolyte membrane to approximate the value of bulk ionomer.

From comparing the experimental findings with available relations for proton conductivity, a clear shortcoming can be identified. Whereas both the Bruggeman relation and percolation theory treat the CCL as an ideal random medium and do not address partial order in the ionomer morphology, the geometry-based relation proposed by Liu et al. cannot fully capture variations in the ionomer morphology. To bridge this gap and reveal essential structural elements in ionomer morphology, the CCL microstructure can be studied using pore-scale simulations.

### Image-supported modeling: assisting the analysis of ionomer morphology

The simulation of material microstructures dates back to the 1970s and 1980s when Joshi^53^ and Quilibier^54^ pioneered the fundamentals of Gaussian processes for stochastic image generation. These early works already outlined the potential of simulations on generated 3D material images to extract transport properties. Stochastic image generation has been applied to PEFC catalyst layers, where multiple works have focused on gas and/or vapor transport through the pore space^55–59^. In the following, we will briefly review works that include simulation of proton conductivity of the electrolyte phase.

Sui et al.^60^ employed a catalyst layer imitation consisting of spherical carbon particles that were randomly placed and fully coated by an ionomer film of uniform thickness. Since the authors aimed to optimize the CCL composition, the single parameter of interest was the ionomer volume fraction of the CCL. Variations of ionomer morphology were not addressed. Nonetheless, this pioneering work demonstrated how relations derived from pore scale simulations can form the basis for cell optimization with basic electrode models.

In the time between 2008 and 2012, multiple works developed more sophisticated simulation approaches of proton transport properties. Hattori et al.^61^ used randomly placed carbon particles and varied the microscopic ionomer thickness and coverage by allowing randomness in the distribution of the ionomer film. They did not evaluate the proton conductivity but calculated the polarisation curve directly through simulation of reactive transport in the 3D model system. From studying variations in ionomer distribution, uniform films with high coverage were found to be most favorable to yield high cell performance. This was rationalized by a favorable connectivity of the electrolyte phase and a high catalyst utilization.

Kim and Pitsch^62^ aimed to refine stochastic structure generation and proposed a sphere-based annealing method, which built on a Gaussian field and used two-point correlation functions from experimental CCL images. They found the conductivity to follow a power law with an exponent of 2. Below an ionomer volume fraction of 0.25, simulation results were seen to deviate from the power law. However, the authors did not interpret these results in terms of a percolation threshold.

To describe the incremental self-assembly of the CCL structure during ink stage, Siddique et al.^63^ proposed an alternative technique for image generation. At first, random seeds were placed in a volume of $200~\text{nm} \times 100~\text{nm} \times 100~\text{nm}$ with 2 nm resolution. Carbon particles agglomerated around the seed points. Subsequently, ionomer was iteratively aggregated around seeds on the carbon particles surfaces. A variation of the I:C ratio revealed a dramatic discrepancy between the Bruggeman relation and simulation results, but the results closely matched the percolation law with percolation threshold of 0.2.

Lange et al.^64^ developed an algorithm based on randomly placed spheres and widely varying geometric parameters, such as particle radius and overlap tolerance. Bigger particles and larger overlap, i.e., less tortuous geometries with less curvature of the ionomer film, led to an increased proton conductivity. Thus, the curvature and roughness of agglomerates must be considered as a source of error when running simulations of transport in thin films. In a follow-up work, Lange et al.^65^ considered several reconstruction algorithms that did not reveal a significant impact on effective transport properties in most cases. In contrast, the results showed a significant dependence on the assumptions about the ionomer morphology, i.e., whether ionomer uniformly covers catalyst agglomerates or randomly aggregates throughout the catalyst layer.

More recently, Inoue et al.^66^ modeled the aggregation of primary carbon particles using an algorithm that emulates particle attraction and repulsion. Two distinct ionomer morphologies were simulated: carbon aggregates coated with ionomer of uniform thickness and partial coverage; and heterogeneously distributed ionomer, where the ionomer resembled a wetting liquid that formed menisci with uniform curvature. Both cases exhibited a power-law behavior at low I:C ratios $\le 0.5$ with an exponent of 1.8. In the case of uniform curvature, a different power-law was observed with an exponent of 3 at I:C $>0.5$, which was linked to inter-agglomerate bridging of the ionomer phase. Since ionomer coverage evolved over I:C ratio in a non-linear manner, the impact of different ionomer film thickness, coverage and content could not be fully disentangled.

The question arises whether the ionomer structure can be resolved through reconstruction from experimental tomographic images. Lange et al. demonstrated that a resolution of 2 nm in a cubic volume of $(200~\text{nm})^3$ gives accurate results for finite-element steady-state proton transport simulations at reasonable computational costs^64,67^. 3D imaging techniques like nano-CT^21,68^ and FIB-SEM^55,67,69,70^ reach a resolution of 10 to 100 nm, which is sufficient for visualizing the ionomer morphology on the agglomerate level, but cannot reliably resolve finer details, such as ionomer film thickness. Additionally, carbon and ionomer have similar densities, thus only a binary image resolving solid and void space can be obtained. However, exchanging the protons of the sulfonic headgroups with Cs$^+$ ions creates sufficient contrast to resolve the ionomer within the CCL. Using this technique, Komini Babu et al.^21^ reported images from CCLs with three different ionomer loadings ($35~\text{wt}\%$, $50~\text{wt}\%$, $60~\text{wt}\%$) and observed changes in ionomer morphology that affected the proton conductivity in simulations and the cell performance in experiments. Images that resolved voxels of $16~\text{nm}^3$ were used to run simulations of proton transport, partially resolving the fine structure of agglomerates and thin films. At the lowest ionomer content, poorly connected aggregates formed, whereas a thick uniform film on the catalyst aggregates formed with increasing amount of ionomer. In a recent study, Goswami et al.^71^ applied a similar approach to PEFC catalyst layers degraded by carbon corrosion, extending the perspective on ionomer morphology to be subject to changes during the cell life-cycle, which consequently might evoke changes in proton conductivity during operation.

Simulations based on experimentally obtained images allow studying only a few points in the parameter space of ionomer morphology. Works discussed above have demonstrated that generating synthetic 3D images provides an alternative pathway to investigating the ionomer morphology since a wide range of structural parameters can be simulated. However, a systematic study on the relation between ionomer structure and proton transport could not be found in the literature.

In this work, we have developed analytical relations between ionomer morphology and proton transport properties in CCLs. To first understand how the CCL microstructure affects proton transport, we have applied stochastic image generation for a wide range of ionomer coverage and film thickness and simulated the effective proton conductivity. Subsequently, the complexity of simulation results has been reduced by resorting to percolation theory to yield analytical relations between decisive structural features in ionomer morphology and the effective proton conductivity of the CCL.

## Model and methodology

This work describes the structure of a conventional CCL, in which primary Pt/C particles aggregate and are partially covered by a skin-like ionomer film. The coverage and thickness of the ionomer film can vary significantly, albeit being accessible in experiment^72,73^, rendering them suitable parameters for modelling works.

The model distinguishes two pore size domains, following the classification from previous modeling works^6, 10,74,75^. The carbon support contains a certain volume fraction of primary pores (1 to 10 nm diameter), whereas secondary pores (10 to 100 nm diameter) form the space between agglomerates^76^. The ionomer is deposited at the agglomerate interface in secondary pores and is assumed to be excluded from primary pores since the ionomer macromolecules are too big to enter.

**Figure 1:**
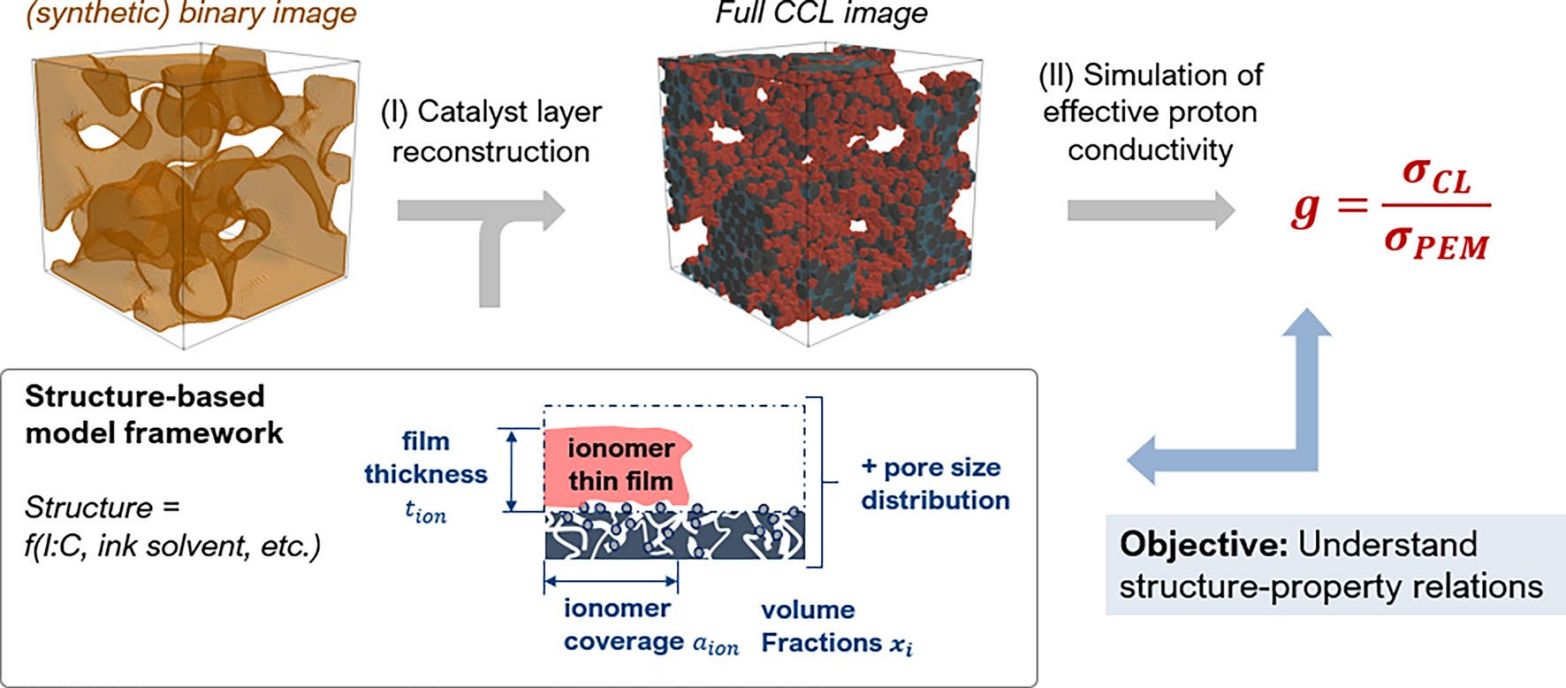
The image generation workflow of the model builds on binary tomografic images, which are combined with information from a structure based model^78^. Calculation of proton conductivity is performed subsequently with the objective to understand underlying structure-property relation of the CCL material.

Correlations between the proton conductivity and the ionomer morphology have been established in two steps (see Fig. 1). First, a 3D image of the CCL structure has been generated based on a synthetic binary image. The phases of ionomer, carbon and pore space have been stochastically reconstructed. The ionomer morphology depends on ionomer coverage and thickness. Next, a steady-state finite-element simulation of proton conductivity within the ionomer phase has been conducted and percolation properties have been evaluated. We have used PoreSpy, an open source software package for Python^77^, to run image generation, manipulation, evaluation and conductivity simulation. All functions and routines referred to in the following are included in PoreSpy unless denoted otherwise. To support the analysis of the simulation results, we have used a composition model for the CCL from our previous work^78^.

### CCL image generation

Image generation begins by creating a binary image that distinguishes between a condensed agglomerate phase, which lumps together carbon, catalyst, primary pores and ionomer, and the secondary pore space. In an operational catalyst layer, water will condense in primary pores. Depending on the operational conditions, the secondary pores can be flooded as well. Water renders the catalyst layer active in the first place, as it serves as reaction medium and proton shuttle. The ionomer also absorbs water, which mobilizes protons provided by the ionomer, thus enabling a high proton conductivity. Therefore, we consider the ionomer phase to include water. Bulk-like liquid water condensed in primary and the secondary pores is not resolved, since it only marginally contributes to the proton conductivity as the proton concentration is several orders of magnitude lower compared to the ionomer electrolyte phase^79^.

Cubic volume elements were used with a length conversion factor of $1~\text{vx} = (2~\text{nm})^3$, adopting the proposed resolution from Lange et al.^64^, which was found to give numerically convergent results also in this work (see Fig. S1 in the supplementary material). Binary tomographic images of CCL materials reported in the literature resemble random heterogeneous media in which the local thickness of the solid phase exhibits a Gaussian size distribution^70,80,81^, with no additional stochastic features. Therefore, our work has employed as well as simple Gaussian field without further features, such as 2-point-correlations. Further, Lange et al.^64,65,67^, demonstrated that the choice of reconstruction algorithm has a limited impact on simulation results, if essential structural features of the CL structure are correctly captured (agglomerated Pt/C particles, ionomer film formation). For this purpose, PoreSpy is the most suitable tool as it allows close control of generated geometries. The reconstruction algorithm presented in the following employs the structural picture of CCL microstructure as supported by the experimental literature^19,76^, consisting of the following assumptions:

1. Carbon support and ionomer form an agglomerates structure.
2. Ionomer does not enter the intra-agglomerate pore space.
3. Ionomer forms a thin film of uniform thickness.
4. The spatial distribution of the solid structure is a Gaussian field, i.e., has no local ordering.
5. Ionomer is randomly distributed in finite-sized building blocks (of ‘patches’) on the agglomerate surface.

Transferring this understanding of CCL microstructure to this work, the PoreSpy software has been used to generate images of agglomerated structures by applying a Gaussian blur filter to a random noise field. This step has been followed by applying a direct threshold to obtain a desired volume fraction of agglomerates $x_{agg}$. The resulting ‘agglomerate phase’ served as a template for the reconstruction of the agglomerated structure. This procedure has been implemented as the blobs function in PoreSpy. The standard deviation $\sigma _b$ for the kernel of the Gaussian filter was adjusted to match the desired average agglomerate size $r_{agg}$, which is controlled by the input parameter *B* in the blobs function,

1 $$\begin{aligned} \sigma _b&= \frac{L}{ 40~B} \quad \text {and } \end{aligned}$$

2 $$\begin{aligned} B&= \frac{L}{ 40 ~ r_{agg} ~ (1-\ln (1-x_{agg}))} \text {.} \end{aligned}$$

**Figure 2:**
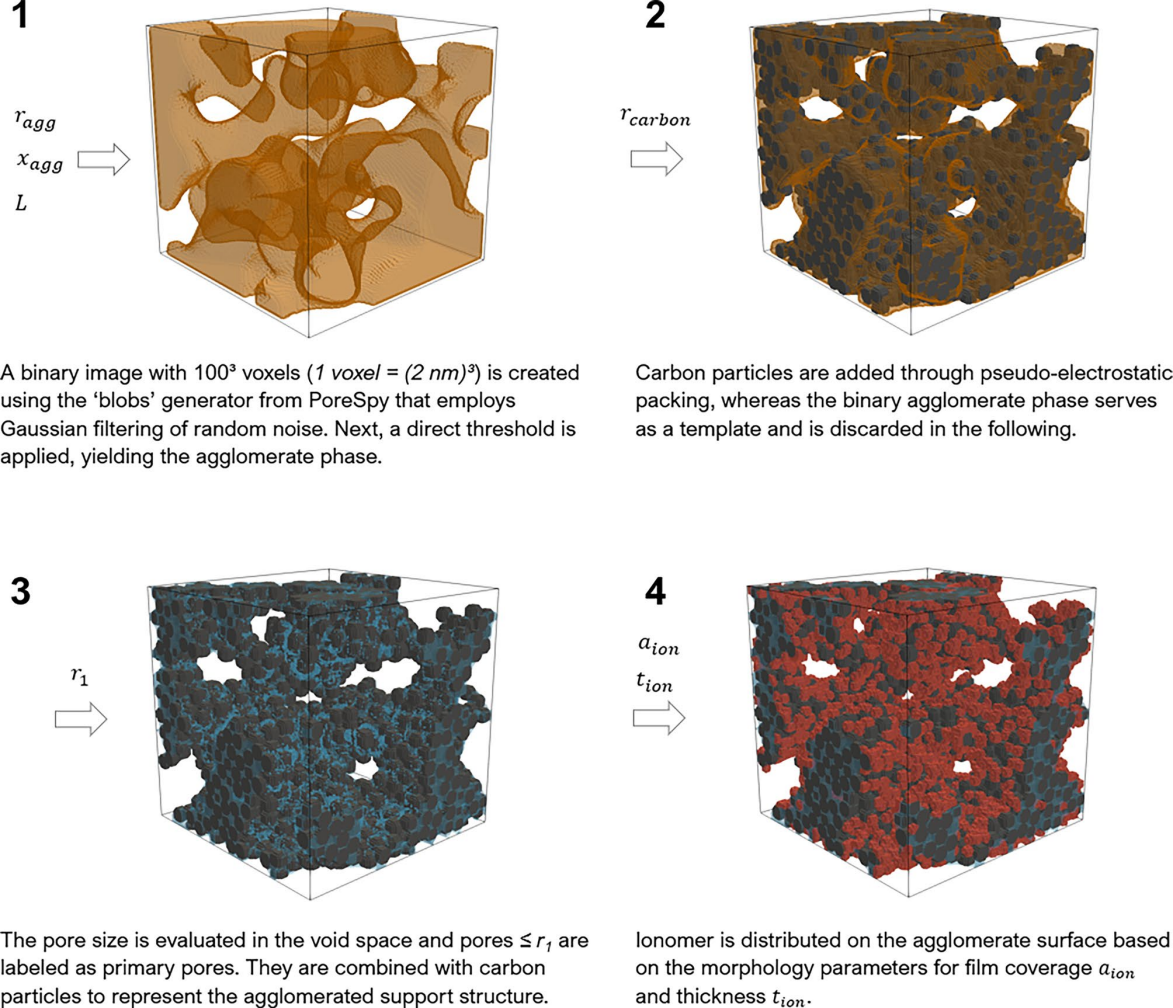
The workflow for image generation follows four steps: (**1**) generation of a binary image, based on tomographic data, (**2 + 3**) reconstruction of the agglomerate structure, resolving carbon particles and primary pores, and (**4**) deposition of ionomer onto the agglomerate surface.

The obtained images were smoothed using binary_opening from the scipy.ndimage package and a sphere with with a radius of $\text{3 vx}$ as structural element. An exemplary image is depicted in Fig. 2(1).

To reconstruct the internal structure of agglomerates, spherical carbon particles with radius $r_{carbon}$ have been placed inside the agglomerate phase (see Fig. 2(2)). The routine pseudo_electrostatic_packing has been used to fill the agglomerate space with carbon particles. This packing algorithm mimics the effect of an electrostatic field pulling particles to the inside of the agglomerate phase. Running an Euclidean distance transform over the agglomerate phase returns a scalar value for each voxel that is interpreted as a measure for the ‘field strength’. Carbon particles have been iteratively placed at the point of maximum field strength that is not occupied by other particles. For further details of the algorithm we refer the reader to the documentation of PoreSpy^77^. Carbon particles were allowed to overlap by a third of the carbon particle radius and protrude into the secondary pore space by half of their radius. This procedure of reconstructing the carbon particles was adopted from a preceding work of Sadeghi et al.^82^. To reconstruct the primary pore space, the space between carbon particles was filtered for pore size using local_thickness and applying a threshold $r_1$. Pores below that threshold are defined as primary pores and lie entirely inside the agglomerate phase (see Fig. 2(3)). All remaining void space between the agglomerates is defined as secondary pore space. In the following, the combined phase of carbon particles and primary pores replaces the agglomerate phase, rejecting the template agglomerate phase from the image. This distinction of primary and secondary pores in the phase image serves to identify the deposition spots of ionomer on the interface of agglomerate and secondary pore space.

The deposition of ionomer on the outer surface of agglomerates employs mathematical morphology operations for binary images provided by the sciPy package^83^. A binary dilation of one voxel has been applied to the agglomerate phase, defining the agglomerate surface. From the resulting set of voxels, a random position was picked to place a piece of ionomer. An ionomer patch was generated from the binary product of a sphere with radius $r_{ion}$ and the set of voxels identifying the agglomerate surface. An ionomer ‘patch’ was then created by running another binary dilation on the ionomer film thickness $t_{ion}$ on the picked subset marking the ionomer surface element to be covered with ionomer. The resulting building block of ionomer was added to the ionomer phase. The result is exemplary illustrated in Fig. 2(4). Through summation of the voxels in the intersection of the ionomer phase and the voxels identifying the agglomerate surface, the ionomer coverage $a_{ion}$ has been estimated. It is defined as the ratio of surface area covered by ionomer to the agglomerate surface,

3 $$\begin{aligned} a_{ion}&= \frac{A_{covered}}{A_{agg}} {\textbf {.}} \end{aligned}$$

The process has been repeated until a predetermined ionomer coverage was reached.

The final image was checked for consistency by evaluating the volume fraction of each phase and the size distribution of pores, agglomerates and ionomer. The volume fractions have been calculated as the ratio of the sum of the voxels in one phase to the total voxel count of the image,

4 $$\begin{aligned} x_i&= \frac{ \sum \texttt {vx}_i }{ L^3} \text {.} \end{aligned}$$

The size distributions of pores, ionomer and agglomerate were obtained from running the function local_thickness on each phase which assigns each voxel the value of the largest sphere that could fit in the local pore that includes the voxel. The function pore_size_distribution returns the histogram data for the distribution of pore sizes, ionomer film thicknesses and agglomerate sizes. Finally, the image was saved to a .vtk file and visualized in ParaView. All parameters used for the image generation workflow are listed in Table 1.

Ionomer coverage and ionomer film thickness are the most sensitive parameters and are of primary interest in this work. All other parameters were tested for their sensitivity as well, as reported in Fig. S2 in the supplementary material.

**Table 1:** Base case parameters for stochastical CCL image generation.

Parameter	Value	Unit
*L*	200	nm
$x_{agg}$	0.5	–
$r_{agg}$	20	nm
$r_{carbon}$	10	nm
$r_1$	6	nm
$r_{ion}$	10	nm
$a_{ion}$	0.5	−
$t_{ion}$	10	nm

### Calculation of proton conductivity

The proton conductivity of the obtained CCL images has been evaluated by solving the following steady-state equation in voxel domains occupied by ionomer,

5 $$\begin{aligned} \nabla \cdot (- \sigma _{ion} \nabla \phi )&=0 \text {.} \end{aligned}$$

The function tortuosity_fd was used to compute the proton conductivity through the ionomer phase using a finite difference approach, similar to the widely used TauFactor software^84^. The function operates by applying boundary conditions automatically for a specified direction, i.e., proton flux enters the volume through one face of the cube and leaves through the opposite face. At the inlet and outlet face of the cube a constant potential and all other faces a zero-flux condition was applied,

6 $$\begin{aligned}&\phi _{in} = const., \quad \phi _{out} = const. \quad \text {with} \quad \phi _{in} > \phi _{out} \quad \text {and} \end{aligned}$$

7 $$\begin{aligned}&{n_\perp } \cdot \nabla \phi = 0 \text { at all other faces.} \end{aligned}$$

The function returns the relative conductivity *g*,

8 $$\begin{aligned} g&= \frac{\sigma _{CL}}{\sigma _{PEM}} \text {,} \end{aligned}$$

which is normalized to the conductivity of the polymer electrolyte membrane, $\sigma _{PEM}$, a known property^4,52^. By using a dimensionless proton conductivity, this work aligns with previous works^4,85^ and allows comparing different operating conditions and ionomers with different molecular structure, ion exchange capacity or molecular weight.

### CCL composition model

To discuss the evolution of ionomer morphology over a range of I:C ratios, we applied the composition model developed previously in Ref.^78^. It accounts for the dependence of ionomer morphology on ink and process parameters via the initial film thickness, $t_0$, and a dispersion parameter, $k_A$. The relations for ionomer film coverage and thickness are

9 $$\begin{aligned} a_{ion}&= 1- \exp \left( - k_A x \right) \quad \text { and} \end{aligned}$$

10 $$\begin{aligned} t_{ion}&= \frac{V_{2,0} }{A_2} \frac{1- \exp ( -k_V \, x )}{1- \exp ( -k_A \, x ) } \text {,} \end{aligned}$$

where *x* denotes the dimensionless ionomer volume per secondary pore volume in an ionomer-free reference sample,

11 $$\begin{aligned} x&= \frac{m_{I:C}}{\rho _{ion} V_{2,0}} \end{aligned}$$

and $k_V$ depends on $t_0$ and the secondary pore space geometry via $A_2$ and $V_{2,0}$,

12 $$\begin{aligned} k_V&= t_0 k_A \frac{ A_2 }{V_{2,0}} \text {.} \end{aligned}$$

As the ionomer film partially occupies the secondary pore space, the remaining ‘free’ secondary pore volume is obtained as

13 $$\begin{aligned} V_{2,free}&= V_{2,0} \left( 1- \exp ( -k_V \, x )\right) \text {.} \end{aligned}$$

The volume of the ionomer film is given by

14 $$\begin{aligned} V_{ion}&= \frac{ A_2 }{V_{2,0}} a_{ion} t_{ion} \text {.} \end{aligned}$$

The difference between film volume and total ionomer volume used to fabricate the CL,

15 $$\begin{aligned} V_{ex}&= \frac{m_{I:C}}{\rho _{ion}} - V_{film}\text {,} \end{aligned}$$

constitutes ‘excess’ ionomer, which forms large aggregates. Volume fractions are defined as follows to perform the analysis of the CL composition,

16 $$\begin{aligned} x_i&= \frac{V_i}{\sum _i V_i} \quad \text {with } i \in \{2,~agg,~ion,~ex\} \text {.} \end{aligned}$$

Here, the agglomerate volume is a sum over carbon and primary pores,

17 $$\begin{aligned} V_{agg}&= 1 / \rho _{carbon} + V_1 \text {.} \end{aligned}$$

The fraction of total ionomer volume $x_{ion,total}$ is given as the sum of ionomer film volume and excess ionomer volume,

18 $$\begin{aligned} x_{ion,total}&= x_{ion} + x_{ex} \text {.} \end{aligned}$$

Table 2 lists values used for the parametrization of the composition model are listed.

**Table 2:** Parameters to predict the ionomer morphology as a fuction of ionomer content.

Parameter	Value	Unit
$V_{2,0}$	0.8	$\text{cm}^3/\text{g}_{carb}$
$V_{1}$	0.2	$\text{cm}^3/\text{g}_{carb}$
$A_2$	20	$\text{m}^2/\text{g}_{carb}$
$t_0$	{4, 6, 8}	nm
$k_A$	{2, 3, 5}	−
$\rho _{ion}$	2	g/cm^3^
$\rho _{carbon}$	2	g/cm^3^

The parameters $t_0$ and $k_A$ allow analyzing the impact of ink processing.

### Extraction of reference data from the literature

We collected literature data for proton conductivity of CLs from various sources listed in Table 3. If reported, the respective value of $\sigma _{PEM}$ was adopted as the reference value for the dimensionless proton conductivity *g*. If not reported, the proposed correlation for PEM conductivity from Gerling et al. ^4^ was applied to obtain the value of $\sigma _{PEM}$ for the respective operating conditions, i.e., relative humidity and temperature, used in a particular literature source,

$$\begin{aligned} R_{PEM}&= 1.2~RH^{-1.44} \exp \left( \frac{ 7.0~\text{kJ}~ \text{mol}^{-1}}{ R T} \right) ~ [\text{m}\Omega ~\text{cm}^2] \quad \text {and} \\ \sigma _{PEM}&= \frac{ 18~\upmu \text{m}}{R_{PEM}} \text {.} \end{aligned}$$

Liu et al. ^52^ demonstrated that for a correct description of the dependence of *g* on $x_{ion}$, the water uptake and the swelling of ionomer need to be taken into account. Thus, if not already done in the respective source, we calculated the wet ionomer volume fraction $x_{ion}$ from the data for the dry ionomer volume fraction $x_{ion,dry}$, using the correlations proposed by Liu et al.^52^,

19 $$\begin{aligned} \lambda&=\left[ 1+0.2325~RH^2~\frac{T-303 K}{303 K} \right] (14.22~RH^3 - 18.92~RH^2 + 13.41~RH) \end{aligned}$$

20 $$\begin{aligned} x_{ion}&= x_{ion,dry} \left( 1 + \frac{\rho _{ion,dry}}{\rho _W} \frac{\lambda M_W}{EW} \right) \text {,} \end{aligned}$$

where $\rho _W$ and $\rho _{ion,dry}$ denote the densities of water ($\approx 1~{\text{g}} \, {\text{cm}}^{-3}$) and dry ionomer ($\approx 2~{\text{g}} \, {\text{cm}}^{-3}$), and $M_W$ is the molecular weight of water ($= 18~{\text{g}} \, {\text{mol}}^{-1}$). For the EW the reported value of the ionomer value was applied. If not reported, $EW = 1000~{\text{g}} \, {\text{mol}}^{-1}$ was assumed.

In some references, only the I:C ratio was reported. In such cases, the ionomer volume fraction was estimated from other CCL data, e.g., CCL thickness, mass loading and densities of ionomer and carbon or the CCL porosity.

Typically, the proton conductivity can be determined from electrochemical impedance spectroscopy (EIS) measurements. The CCL proton conductivity is extracted from EIS spectra by fitting a transmission line model or equivalent circuit model. However, some sources obtained the proton conductivity through DC measurements. These different methods can give diverging results under low humidity conditions. Qi et al.^85^ proposed that AC measurements, such as EIS, include more tortuous and dead-ended pathways. Under humid conditions, the discrepancy is diminished as condensated water provides bridging pathways that lower the apparent tortuosity. High humidity was considered in all data reported here, thus a divergence between AC and DC methods is not suspected in the dataset.

**Table 3:** Sources in literature for experimental data correlating CCL proton conductivity with ionomer content.

Reference	Method to measure $\sigma _{CCL}$	Reference value for $\sigma _{bulk}$	Ionomer content information	Relative humidity *RH*	Temperature *T*	Ionomer used
Liu et al.^52^	EIS	Provided as *g*	As $x_{ion}$	$35\%, 50\%,$ $75\%, 122\%$	80 °C	Nafion 1050 EW, Nafion 850 EW
Yakovlev et al.^12^	EIS	120 mS cm^−1^, estimated	As I:C	$100\%$	60 °C	Nafion 1100 EW
Boyer et al.^44^	DC	100 mS cm^−1^, provided	As $x_{ion}$	$100\%$	50 °C	Nafion 960 EW
Suzuki et al.^86^	EIS	89 mS cm^−1^, estimated	As $x_{ion}$	$100\%$	Room temperature	Nafion, no EW reported
Du et al.^87^	DC	300 mS cm^−1^, provided	As $x_{ion}$	$100\%$	80 °C	Nafion, no EW reported
Modestov et al.^79^	EIS	7 mS cm^−1^, provided	As I:C	$100\%$	Room temperature, 22–25 °C	Nafion, no EW reported
Havránek and Wipperman^88^	EIS	120 mS cm^−1^, provided	As $x_{ion}$	Flooded	40 °C	Nafion, no EW reported

## Results

In the following, firstly, we review various sets of literature data and discuss the observed trends. Secondly, we present a parameter study to understand these trends and identify the essential structural elements that determine the proton conductivity. Thirdly, we derive a modified approach based on percolation theory that accounts for insights from simulations of proton conductivity. In the fourth step, implications of our results for CCL fabrication and the prospects of the approach to guide the design of CCLs with tailored properties will be discussed.

### Literature data and classical relations for proton transport

**Figure 3:**
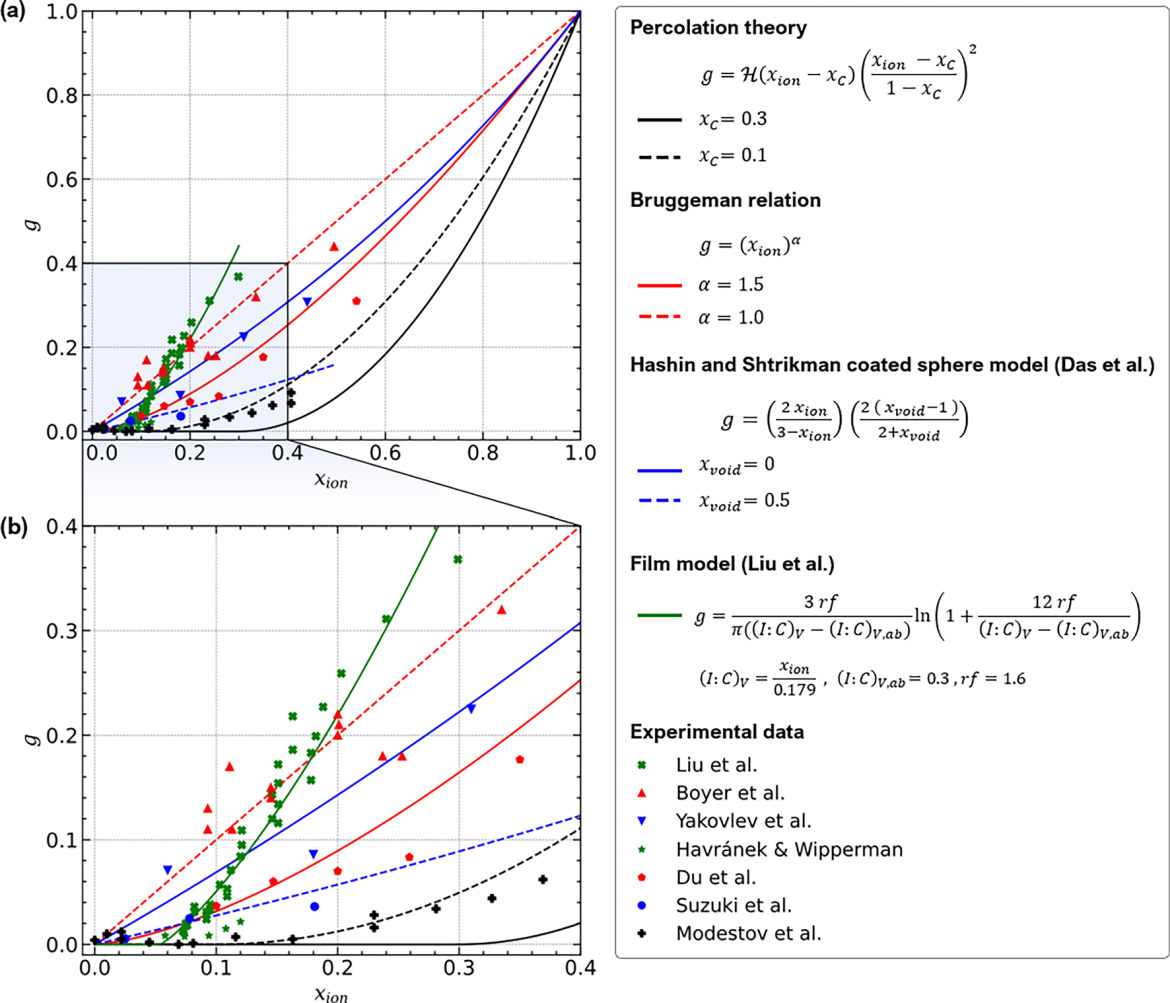
Comparison of literature data^12,44, 52,79,86–88^ for proton conductivity and available relations for heterogeneous random media (Bruggeman relation, percolation theory, Hashin and Shtrikman coated sphere model proposed by Das et al.^48^ and spherical film model proposed by Liu et al.^52^). Please note that the original equation provided in Ref.^48^ has been rearranged for the sake of clarity.

The selected datasets for ionomer content $x_{ion}$ and relative conductivity *g* are plotted in Fig. 3, together with relations proposed in the literature. The Bruggeman relation with an exponent of $\alpha = 1$ roughly fits the data from Boyer et al.^44^, which also sets the limit for the highest reported values of relative conductivity. Assuming that the proton conductivity of the CCL should not be higher than $\sigma _{PEM}$, values appearing significantly above this limit should not be possible. Other datasets (Suzuki et al.^86^, Yakovlev et al.^12^, Havránek and Wipperman^88^, Du et al.^87^) scatter around a classical Bruggeman relation with $\alpha = 1.5$.

Datasets of Liu et al.^52^, Havránek and Wipperman^88^, and Modestov et al.^79^ are compatible with a percolation threshold of $x_c \approx 0.1$. The dataset from Modestov et al.^79^ follows a percolation law with this percolation threshold and a critical exponent of 2. The thin-film model of Liu et al.^52^ describes their own dataset well. However, the model is physically inconsistent since it predicts relative conductivities $g > 1$ for $x_{ion} \rightarrow 1$ , where as *g* must approach 1 from below.

Overall, the cited literature data do not exhibit a unique relation between *g* and $x_{ion}$. The reason might be difference in fabrication approaches and conditions that result in different ionomer morphologies.

### Simulation results

**Figure 4:**
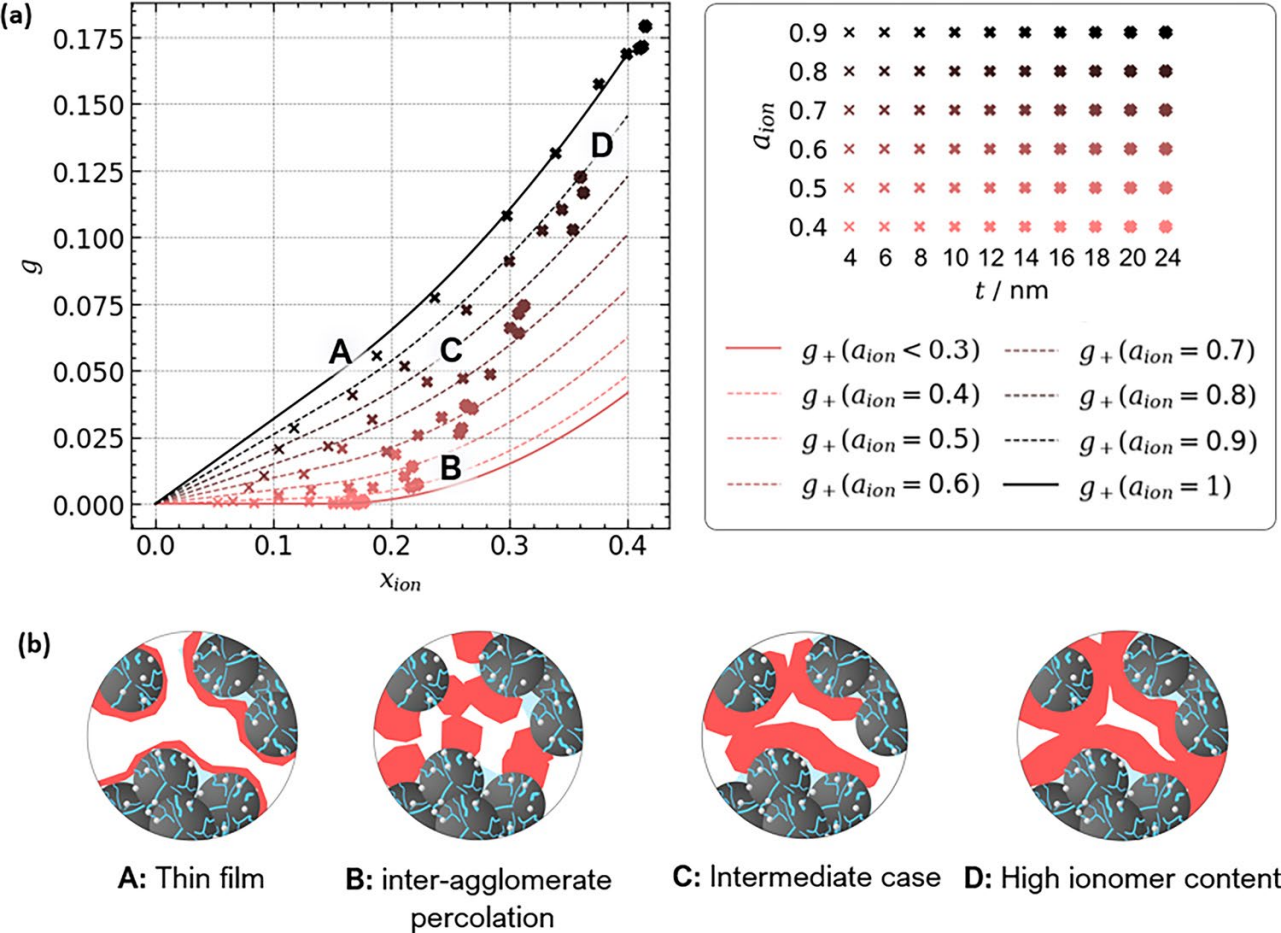
(**a**) Simulation results from variation of ionomer coverage $a_{ion}$ and thickness *t*. The agglomerate volume fraction was held constant ($x_{agg} = 0.5$). The proposed relation based on percolation theory is plotted as well. (**b**) Four cases of ionomer morphology can be identified and their structural images are illustrated.

To unravel the relations between proton conductivity and ionomer content, we have conducted a parameter study for ionomer coverage $a_{ion}$ and film thickness $t_{ion}$. The results for *g* are plotted over $x_{ion}$ in Fig. 4a. The highest values for *g* are obtained for morphologies with large ionomer coverage, $a_{ion} > 0.8$. In this case, *g* scales almost linearly in both *t* and $x_{ion}$ for small ionomer contents. Extrapolation towards $x_{ion} = 0$ indicates a vanishing percolation threshold. Please note that our simulations could not cover the range $0~\text{nm} \le t_{ion} < 4~\text{nm}$ due to the limited resolution of the generated images. A film represented by only one voxel in thickness would disconnect on the curved surface of the agglomerates and would give a non-physical numerical fragment. Additionally, films thinner than 5 nm have been rarely observed in literature. Hence, the resolution chosen in this work (2 vx or 4 nm) can be considered adequate.

A different picture emerges for scenarios where $a_{ion} < 0.5$. A percolation threshold is evident around $x_{ion} \approx 0.15$. Below this threshold, the simulation results indicate very low proton conductivity. Where the threshold is exceeded, the conductivity scales with a power-law behavior with exponent $\alpha \approx 2$. The intermediate cases of moderate ionomer coverage interpolate between the limiting cases of high and low ionomer coverage.

The underlying structural pictures explaining the simulation results are illustrated in Fig. 4b (A)–(D). In the case of a thin film (A), percolation on the agglomerate surface governs proton conductivity. Oppositely, at low coverage and high thickness, randomly placed coarse ionomer pieces connect (B), leading to a situation that resembles 3D continuum percolation. The intermediate cases (C) have a moderate ionomer coverage, thus percolation on the agglomerate surface plays a role, but also some random connectivity of ionomer across the inter-agglomerate space occurs. All three cases converge with increasing amount of ionomer into case (D). If all volume not already occupied by Pt/C agglomerates is filled with ionomer no variation in the morphology is possible, hence *g* converges into a single value, as observed in the simulation results.

**Figure 5:**
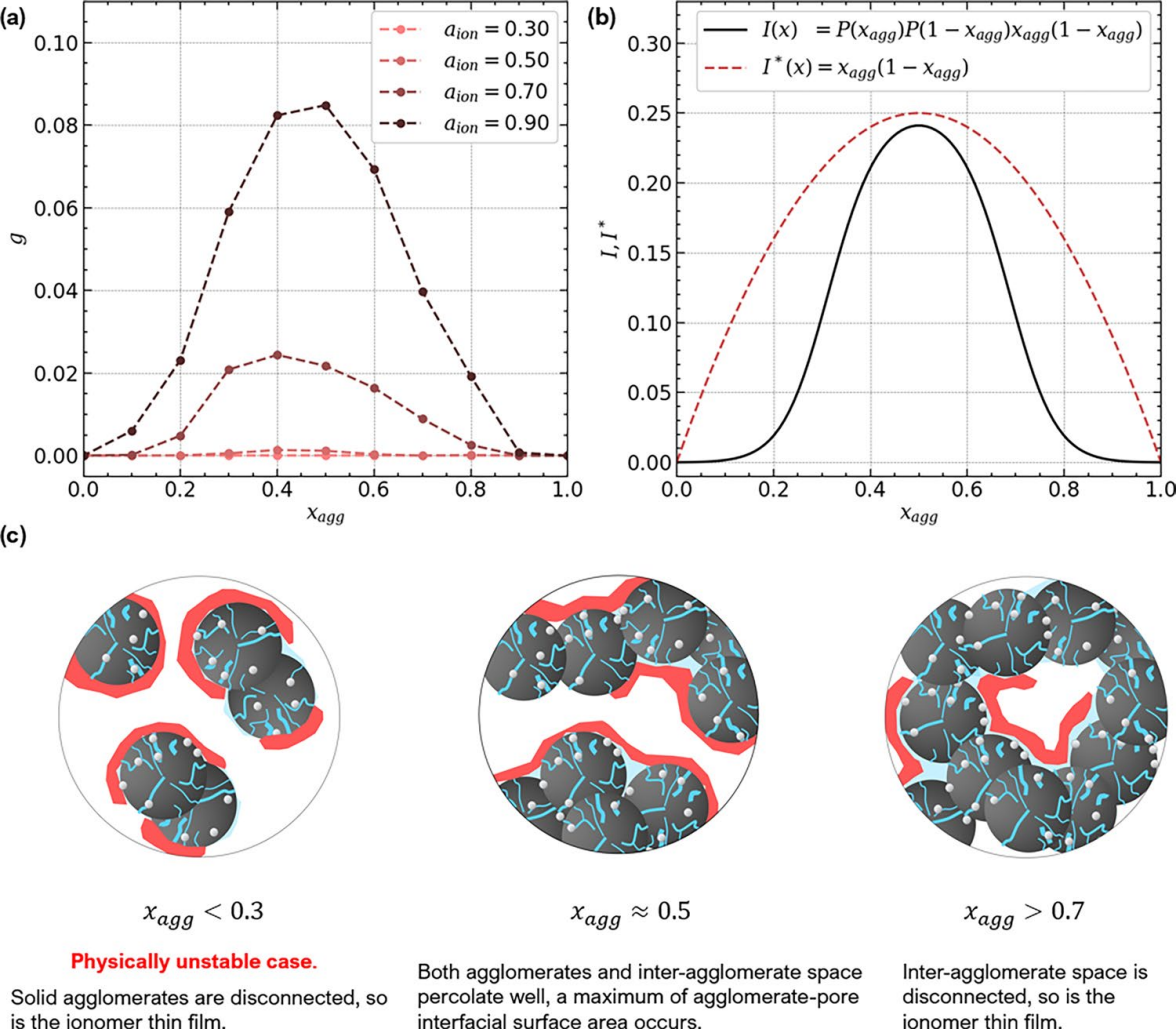
(**a**) Simulation results of proton conductivity for the variation of $x_{agg}$ and ionomer coverage $a_{ion}$. Ionomer film thickness was held constant ($t = 10~\text{nm}$). (**b**) The observed trend can be described using Eq. (23) for the percolation interfacial surface area between agglomerates and secondary pores. (**c**) The underlying structural images are illustrated.

To analyse the impact of the support structure, in Fig. 5a the volume fraction of the agglomerate, $x_{agg}$, was varied between 0.1 and 0.9, while the ionomer film thickness was held constant at $t_{ion} = 10~\text{nm}$. The simulation was repeated for $a_{ion} = \{0.4,~ 0.6,~0.7,~0.9\}$. For $x_{agg} = 0.3~...~0.7$, secondary pore space is mostly open, i.e., pathways reaching through the whole CL exist. In the same range, the agglomerate phase is continuous as well, as illustrated in Fig. 5c. If both phases provide continuous pathways, then the surface of the agglomerate-pore interface is also well-connected and allows pathways for proton conductivity across the CL. Note that a structure with $x_{agg} < 0.3$ would be physically unstable, since agglomerates would be largely disconnected.

When $x_{agg}$ exceeds 0.7, secondary pores become closed and disconnected and the proton conductivity declines steeply. Between these two boundaries, *g* exhibits a maximum, whose height increases with $a_{ion}$ above $\approx ~0.4$. Below that value, no closed path for proton transport is formed by the ionomer film deposited on the agglomerate-pore interface. This implies two requirements for high high proton conductivity: not only the agglomerate-pore interface must provide a well-connected network; also the ionomer coverage must be sufficiently high for the ionomer to percolate.

### Analytical relations for proton conductivity

The previous discussion has shown that the proton conductivity depends on the surface density of agglomerate/pore interface per CCL volume. This finding allows to derive a first analytical approach to describe the simulation results. The percolation theory approach for the interface density $I(x_A,x_B)$ in a binary mixture can reproduce the trend in $x_{agg}$ (see Fig. 5b),

21 $$\begin{aligned} I(x_A,x_B)&= x_A P(x_A) x_B P(x_B)\text {,} \end{aligned}$$

where $x_A$ and $x_B$ denote the percolating phases in an binary continuum and *P*(*x*) the percolation probability, represented by a sigmoid function,

22 $$\begin{aligned} P(x) = \frac{1}{1 + \exp \left( \frac{-(x - x_c)}{b} \right) } \end{aligned}$$

with $b = 0.01$ and $x_c = 0.3$. As the conductivity scales with the interfacial surface area between agglomerate and the complementary phase of pores and ionomer, we replace $x_A$ with $x_{agg}$. Thus, $x_B$ becomes $1-x_{agg}$, yielding

23 $$\begin{aligned} g_{film}&\sim I(x_{agg}) = x_{agg} P(x_{agg}) (1-x_{agg}) P(1-x_{agg}) \text {.} \end{aligned}$$

Further, simulation results indicate that for thin films *g* scales with a power-law in ionomer coverage. Therefore, a percolation law for conductivity as a function of ionomer coverage $a_{ion}$ is applied,

24 $$\begin{aligned} g_{film}&\sim \mathcal {H}(a_{ion} -a_c) \left( \frac{a_{ion}-a_c}{1-a_c} \right) ^{2} \text {.} \end{aligned}$$

Here, $\mathcal {H}$ represents the Heaviside step function and $a_c$ the percolation threshold. A linear scaling of *g* with film thickness has been observed in simulations,

25 $$\begin{aligned} g_{film}&\sim t_{ion} \text {.} \end{aligned}$$

Combining Eqs. (23), (24) and (25) yields an analytical relation for the proton conductivity in thin films,

26 $$\begin{aligned} g_{film}&= \tau _{agg} \frac{A_{agg}}{V_{CL}} I(x_{agg}) \mathcal {H}(a_{ion} -a_c) \left( \frac{a_{ion}-a_c}{1-a_c} \right) ^{2} t_{ion} \text {,} \end{aligned}$$

where $\frac {A_{agg}}{V_{CL}}$ is a scaling factor that accounts for the total agglomerate surface per CCL volume. It has the units of an inverse length and depends on agglomerate size. The simulation results for thin films revealed that the surface to volume ratio of a cylindrical geometry can be applied,

27 $$\begin{aligned} \frac{A_{agg}}{V_{CL}}&= \frac{2}{r_{agg}} \text {.} \end{aligned}$$

Of course, the agglomerate phase has no cylindrical geometry. However, when the local thickness is evaluated by finding the largest sphere to fit, one could span an approximately circular perimeter and apply a differential cylindrical volume element with $V = \pi r_{agg}^2 dL$ and $A = 2 \pi r_{agg} dL$.

If the ionomer film is sufficiently thin, $x_{ion}$ can be calculated from $t_{ion}$ and $a_{ion}$,

28 $$\begin{aligned} x_{ion}&= I^*(x_{agg}) \frac{A_{agg}}{V_{CL}} a_{ion} t_{ion} \nonumber \\&= I^*(x_{agg}) \frac{2}{r_{agg}} a_{ion} t_{ion}\text {.} \end{aligned}$$

Here $I^*(x_{agg})$ indicates the same interfacial factor as $I(x_{agg})$ in Eq. (23), but includes the non-percolating interfacial area. Thus, the factors *P*(*x*) and $P(1-x)$ are dropped. Still, $I^* \approx I$ in $x_{agg} = 0.3... 0.7$. Anyway, values outside this range are not of relevance. In a CL with $x_{agg} < 0.3$ the mechanical suppport collapses, thus such a CL is unphysical; at $x_{agg} > 0.7$ the pore volume closes and does not provide percolation pathyways.

The additional factor $\tau _{agg}$ in Eq. (26) accounts for the tortuosity of the curved agglomerate surface. Assuming a spherical curvature of the smoothed Gaussian field used in this work, $\tau _{agg} = \frac {1}{\pi }$ was chosen and matches the simulation results.

When Eqs. (26) and (28) are combined, the factors $I(x)\frac {A_{agg}}{V_{CL}}$ cancel out, yielding an expression for $g_{film}$, which solely depends on $x_{ion}$ and $a_{ion}$,

29 $$\begin{aligned} g_{film}&= \frac{1}{\pi } \mathcal {H}(a_{ion} -a_c) \left( \frac{a_{ion}-a_c}{1-a_c} \right) ^{2} \frac{x_{ion}}{a_{ion} } \text {.} \end{aligned}$$

In scenario (C) of Fig. 4, a thin film does not form and connectivity establishes across the inter-agglomerate space. The volume fraction of the inter-agglomerate space is defined as $x_2 = 1-x_{agg}$. An adapted percolation law can be applied, accounting for the connectivity of ionomer in the secondary pore space,

30 $$\begin{aligned} g_{2}&\sim \mathcal {H}(x_{ion} / x_2 -x_c) \left( \frac{x_{ion} / x_2-x_c}{1-x_c} \right) ^2 \text {.} \end{aligned}$$

In Eq. (30) the ionomer volume fraction in the inter-agglomerate space is decisive for establishing percolating pathways. Further, $g_2$ scales with the connectivity of the inter-agglomerate space,

31 $$\begin{aligned} g_{2}&\sim \mathcal {H}( x_2 -x_c) \left( \frac{x_2-x_c}{1-x_c} \right) ^2 \text {,} \end{aligned}$$

where the inter-agglomerate volume fraction $x_2$ is used to describe percolation across the catalyst layer volume. Merging Eq. (30) with Eq. (31) yields the analytical expression for $g_2$ as a function of $x_{ion}$ and $x_2$,

32 $$\begin{aligned} g_{2}&= \mathcal {H}(x_{ion} / x_2 -x_c) \left( \frac{x_{ion} / x_2-x_c}{1-x_c} \right) ^2 \mathcal {H}( x_2 -x_c) \left( \frac{x_2-x_c}{1-x_c} \right) ^2 \text {.} \end{aligned}$$

If $x_{ion} \rightarrow x_2$, i.e., all secondary pore volume is replaced by ionomer, Eq. (32) converges to a percolation law for random 3D media.

The expressions for $g_{film}$ and $g_2$ describe the limiting cases where solely thin films on the agglomerates or inter-agglomerate connections control the conductivity. However, for the majority of ionomer morphologies, an intermediate behavior will occur, i.e., the contributions from the thin film and inter-agglomerate percolation will superpose in a random network type fashion. Thus, the effective conductivity can be anything between a series of resistors and a parallel circuit. The highest proton conductivity conceivable, $g_+$, is the sum of both contributions, assuming ideal parallel proton transport pathways,

33 $$\begin{aligned} g_+&= g_{film} + g_2 \text {.} \end{aligned}$$

This expression is plotted in Fig. 4 together with the simulation results. The linear dependence for thin films with high coverage can be clearly distinguished. The threshold behavior at low ionomer coverage is well captured. The extend and slope of the linear range in $x_{ion}$ scale with $a_{ion}$ and provide a transition for the full spectra of ionomer morphologies studied in the simulation.

**Figure 6:**
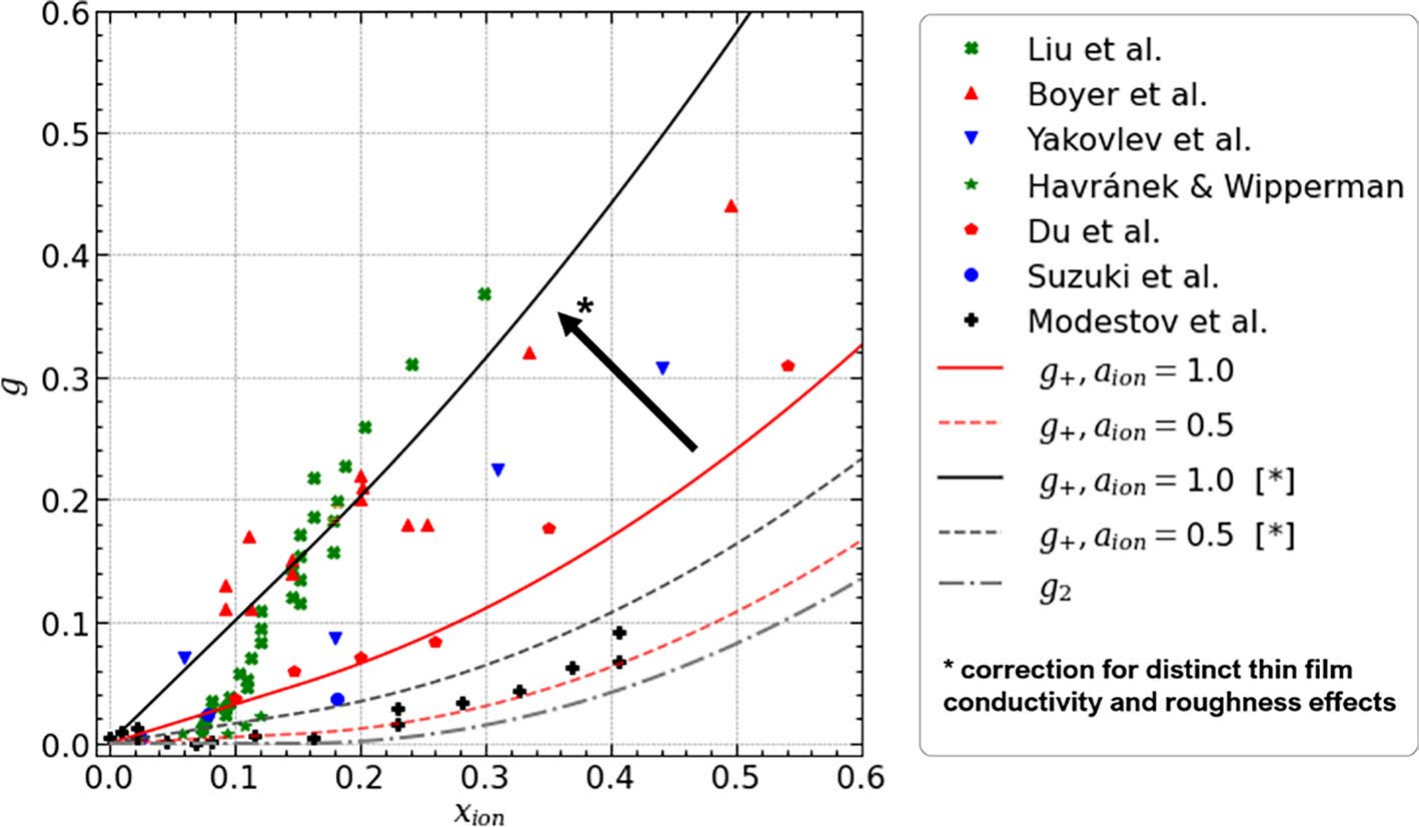
Comparison of literature data for proton conductivity and proposed relations. As a correction for distinct thin film conductivity and roughness effects, the factor $\frac {1}{\pi }$ was dropped in $g_{film}$.

In Fig. 6, $g_+$ was plotted for moderate and high ionomer coverage, $a_{ion} = 0.6$ and 1.0, together with datasets from the literature. The proposed analytical solution for $g_+$ cannot capture the literature data. The deviation is especially large for datasets with high conductivities and a linear trend over $x_{ion}$, e.g., from Boyer et al., indicating that the conductivity of the ionomer thin film is underestimated by the model. As discussed above, agglomerate roughness largely affects proton conductivity. The roughness and curvature in real catalyst layer materials might be significantly lower than in simulations due to water uptake or ionomer swelling^85^ that effectively reduces the tortuosity of the proton conducting pathways. Further, experimental work indicated that the assumption of $g_{ion} \approx g_{bulk}$ might not be true for thin ionomer films. Experimental works reported a lower proton conductivity of PFSA thin films on SiO$_2$ substrates^89,90^ and attributed the impaired proton mobility to the lack of phase-separated water^91^. However, on Pt substrates a well defined water layer at the Pt/ionomer interface forms^92,93^, which boosts conductivity by one order of magnitude compared to SiO$_2$ substrates^94^. Hence, it might be the case that the thin film conductivity in catalyst layers is even higher than the ionomer bulk conductivity. A recent modeling study from the literature evaluated different conceivable molecular structure regimes that might establish on real Pt/C catalyst substrates and found a significant impact on the resulting proton conductivity of the CCL^17^. However, both the effects of tortuosity and distinct ionomer conductivity cannot be quantified reliably. Setting $\tau _{agg} = 1$ in Eq. (26) yields good agreement with the full range of experimental data, hinting that the thin film conductivity in polymer-based catalyst layers is effectively increased by a factor of $~\approx 3$.

The comparison of the corrected relation $g+$ with literature data illustrates how distinct ionomer morphologies can explain different trends in proton conductivity and their dependence on structural parameters, such as $a_{ion}$. Single datasets are well captured by the model and can be linked to different scenarios of ionomer morphology. For instance, the dataset of Boyer et al.^44^ closely follows $g_+(a_{ion} = 1)$. Thus, the observed linearity of *g* in $x_{ion}$ can be explained by a thin film morphology present in the CCL material studied. The datasets of Yakovlev et al.^12^, Suzuki et al.^86^ and Du et al.^87^ align along the relation for the intermediate morphology of moderate ionomer coverage. Some data, e.g., from Modestov et al.^79^, closely follow the relation for low ionomer coverage. Only the dataset of Liu et al.^52^ seems to be difficult to capture by any relation. It is possible that the ionomer morphology largely changes as ionomer content increases, i.e., the ionomer coverage might be a function of $x_{ion}$.

If the approximation $g_+$ is applied to structures with high ionomer content, i.e., $x_{ion} > 0.5$, it might overestimate the proton conductivity. The two pathways for proton conductivity (ionomer thin film and inter-agglomerate percolation) are not separated anymore because the ionomer thin film and larger ionomer pieces in the inter-agglomerate space increasingly overlap. This renders the assumption of two parallel pathways for proton transport invalid, thus the effective conductivity will be lower. However, the proposed approximation accurately describes the simulation results over the catalyst layer composition in the range of technical relevance.

### Adjusting CCL fabrication parameters to tailor proton conductivity

The question arises how ionomer coverage and film thickness depend on the I:C ratio and how the ionomer structure might be tuned during CCL fabrication. Therefore, we have employed a composition model and aimed at providing various references to practical approaches (ionomer content, ionomer dispersion by solvent, carbon surface modifications), which implement the specific modifications of the CCL microstructure.

Experimental studies varying the ionomer content reported that the ionomer coverage increases sharply upon initially adding of ionomer. Subsequently, a growth in film thickness follows^76,95^. At high ionomer contents, excess ionomer aggregates can form that do not contribute to the ionomer film^24–26^. How this resulting ionomer structure can be controlled by ink and process parameters, such as the solvent used, is complex and subject to ongoing research^30,31^. The interactions between Pt/C surface, ionomer and solvent control the self-assembly process during ink stage and largely determine the resulting ionomer morphology. The evolution of $a_{ion}$ and $t_{ion}$ with the I:C ratio can be captured by the composition model developed in Ref.^78^ and allows to discuss the impact of basic ink parameters.

**Figure 7:**
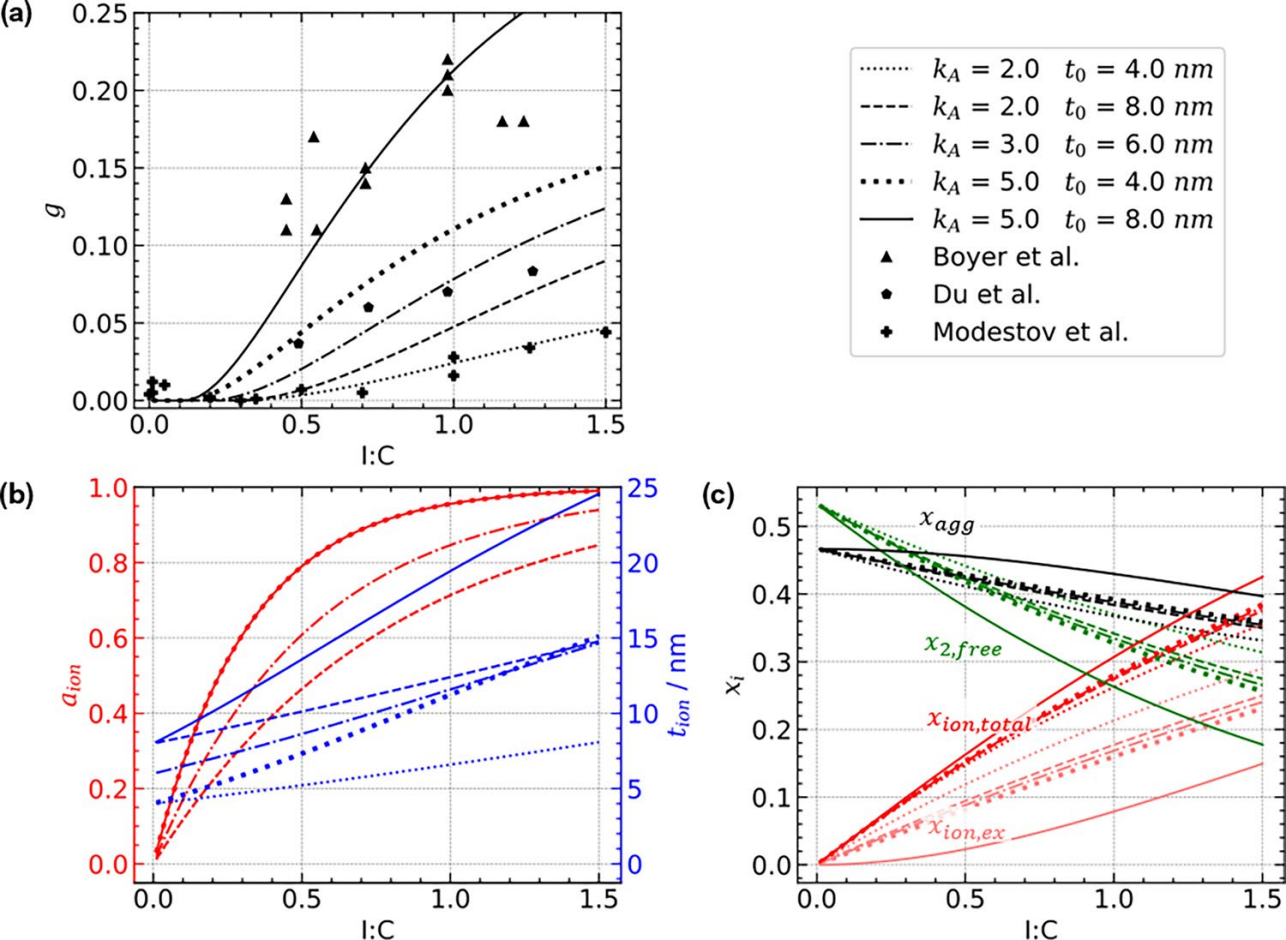
(**a**) Evolution of proton conductivity over I:C ratio, depending on the ink parameters $k_A$ and $t_0$. (**b**) Ionomer coverage and thickness emerge differently for varying ink parameters. Please note that curves of $a_{ion}$ calculated from equal values of $k_A$ always coincide. (**c**) Depending on the ink parameters, also the trajectory of volumetric composition over I:C ratio, e.g., the volume faction of the pore network or aggregates of excess ionomer, is altered.

To link structure formation during ink stage and proton conductivity in the resulting catalyst layer, in Fig. 7 multiple cases for the ink parameters $k_A$ and $t_0$ that we have studied over an I:C range from 0 to 1.5. The proton conductivity predicted by the expression for $g_+$, using the correction discussed above, is plotted together with the literature data from Boyer et al.^44^, Du et al.^87^, and Modestov et al.^79^. The analysis of the ionomer coverage and film thickness (Fig 7b), and volumetric composition of the catalyst layer (Fig. 7c) reveals how the microstructure evolves over increasing I:C ratio in different scenarios. The dataset from Boyer et al. can be described choosing the values $k_A = 5$ and $t_0 = 8~\text{nm}$, i.e., the ionomer adsorbs well on the agglomerate surface and the resulting film is rather thick (>15 nm at I:C >0.6). Two effects contribute to the high proton conductivity in this case: the effective percolation threshold of *g* is lowered, as ionomer coverage increases sharply at low I:C ratios; and the higher film thickness further enhances conductivity, because the proton conductivity increases proportionally with ionomer film thickness.

Reducing ionomer dispersion and initial film thickness ($k_A = 2$ and $t_0 = 4~\text{nm}$) results in a significantly impaired proton conductivity, as reported in the dataset of Modestov et al. Sufficient ionomer coverage is established only at rather high I:C ratios ($a_{ion} > 0.5$ at I:C $> 0.5$) and the resulting film thickness is low, $< 8~\text{nm}$ for the full range of I:C studied. In such a scenario, a major fraction of the ionomer is not dispersed in the ionomer film, but forms larger aggregates, which do not contribute to proton conductivity. As only little ionomer is deposited as part of the thin film in the secondary pore space, more pore volume remains free (see $x_{2,free}$ in Fig 7c).

In intermediate scenarios as for the dataset of Due et al., in which either $k_A$ or $t_0$ is high, or both parameters are moderate (e.g., $k_A = 3$ and $t_0 = 6~\text{nm}$), the volumetric compositions of the catalyst layers are quite similar, because the ionomer film volume is proportional to the product of $a_{ion}$ and $t_{ion}$. However, the proton conductivity varies significantly. Here, the case of a stronger ionomer-carbon interaction ($k_A = 5$) and low initial film thickness ($t_0 = 4~\text{nm}$) results in proton conductivities twice as high as in the opposite case ($k_A = 2$ and $t_0 = 8~\text{nm}$). The gain in proton conductivity from higher ionomer coverage clearly exceeds the gain due to increasing film thickness. This implies measures to adjust the ink formulation or interaction of ionomer with the Pt/C catalyst that foster a thinner film with higher coverage. Efforts in the experimental literature match these findings, such as adjusting solvent composition^24,32–34^ or doping the carbon support with nitrogen containing groups^35,96^ to achieve a higher dispersion of ionomer.

## Conclusion

Polymer electrolyte fuel cells must be designed and optimized to meet the demands of power output and durability. The key to achieving this goal lies in the cathode catalyst layer (CCL) and its microstructure. To this end, we have reviewed modeling approaches for CCL design. We have identified the crucial gap that available relations between composition and proton conductivity in the catalyst layer, such as the Bruggeman relation or percolation theory, neglect specific structural features of the ionomer morphology within the CCL microstructure.

In this work, we have used direct numerical simulations studying the impact of ionomer morphology on proton conductivity. In the past, image-based pore scale simulations focused on gas and liquid transport, but rarely discussed proton conductivity. However, stochastic image generation was found to be a versatile technique to study the impact of different ionomer morphologies. Adopting this approach, we have employed virtual structure generation to generate synthetic binary images that mimic tomographic data, followed by stochastic reconstruction of carbon and ionomer phases. Proton conductivity has been simulated over a wide range of structure parameters in terms of ionomer coverage and film thickness. Two distinct limiting structural regimes could be identified: ionomer can either form a thin film on the catalyst agglomerate surface, where the connectivity of the ionomer coverage dominates the observed trends; or the ionomer forms pieces with substantial thickness that only partially cover the Pt/C agglomerates but connect across the inter-agglomerate space.

Building on these insights from simulation results, a new analytical relation between ionomer morphology and proton conduction was derived from percolation theory. This relation was found to be in close agreement with simulation results and it directly links structural metrics of ionomer coverage and thickness with the proton conductivity. It can capture both limiting cases and reliably interpolates intermediate morphologies. Trends in the literature from multiple sources could be captured. The differences in experimental trends could be linked to variations of ionomer morphology.

To provide guidance for purposeful CCL design and fabrication, structure formation during ink stage has been addressed using a composition model from a previous work. Ionomer morphology and proton transport have been studied over a range of ink parameters, including the I:C ratio, initial ionomer film thickness, and the ionomer dispersion parameter. From this analysis, measures to optimize proton conductivity have been derived and were found in agreement with current experimental literature.

This work demonstrates how profound quantitative modeling and understanding of the relation between CCL microstructure and effective properties can guide the interpretation of experimental data and provide a framework for tailored fuel cell design.

### Supplementary Information

Supplementary Figures.

## Data Availability

The authors declare that the results and data to reproduce the methods and results are reported in full in the article.

## References

[1] Pollet BG, Kocha SS, Staffell I (2019). Current status of automotive fuel cells for sustainable transport. Curr. Opin. Electrochem..

[2] Raistrick, I. D. Modified gas diffusion electrode for proton exchange membrane fuel cells, In *Proceedings of the Symposium on Diaphragms, Separators, and Ion-Exchange Membranes*, Vol. 86, 72, (1986).

[3] Wilson MS, Gottesfeld S (1992). Thin-film catalyst layers for polymer electrolyte fuel cell electrodes. J. Appl. Electrochem..

[4] Gerling C, Hanauer M, Berner U, Andreas Friedrich K (2021). Full factorial in situ characterization of ionomer properties in differential PEM fuel cells. J. Electrochem. Soc..

[5] Huang J, Li Z, Zhang J (2017). Review of characterization and modeling of polymer electrolyte fuel cell catalyst layer: The blessing and curse of ionomer. Front. Energy.

[6] Eikerling M, Kornyshev A (1998). Modelling the performance of the cathode catalyst layer of polymer electrolyte fuel cells. J. Electroanal. Chem..

[7] Wang Q, Eikerling M, Song D, Liu Z, Navessin T, Xie Z, Holdcroft S (2004). Functionally graded cathode catalyst layers for polymer electrolyte fuel cells. J. Electrochem. Soc..

[8] Song D, Wang Q, Liu Z, Eikerling M, Xie Z, Navessin T, Holdcroft S (2005). A method for optimizing distributions of Nafion and Pt in cathode catalyst layers of PEM fuel cells. Electrochim. Acta.

[9] Secanell M, Carnes B, Suleman A, Djilali N (2007). Numerical optimization of proton exchange membrane fuel cell cathodes. Electrochim. Acta.

[10] Eikerling M, Ioselevich A, Kornyshev A (2004). How good are the electrodes we use in PEFC?. Fuel Cells.

[11] Kobayashi A, Fujii T, Harada C, Yasumoto E, Takeda K, Kakinuma K, Uchida M (2021). Effect of Pt and ionomer distribution on polymer electrolyte fuel cell performance and durability. ACS Appl. Energy Mater..

[12] Yakovlev YV, Lobko YV, Vorokhta M, Nováková J, Mazur M, Matolínová I, Matolín V (2021). Ionomer content effect on charge and gas transport in the cathode catalyst layer of proton-exchange membrane fuel cells. J. Power Sources.

[13] Alink R, Singh R, Schneider P, Christmann K, Schall J, Keding R, Zamel N (2020). Full parametric study of the influence of ionomer content, catalyst loading and catalyst type on oxygen and ion transport in PEM fuel cell catalyst layers. Molecules.

[14] Xing L, Shi W, Su H, Xu Q, Das PK, Mao B, Scott K (2019). Membrane electrode assemblies for PEM fuel cells: A review of functional graded design and optimization. Energy.

[15] Wang Y, Ruiz Diaz DF, Chen KS, Wang Z, Adroher XC (2020). Materials, technological status, and fundamentals of PEM fuel cells—A review. Mater. Today.

[16] Suter TAM, Smith K, Hack J, Rasha L, Rana Z, Angel GMA, Shearing PR, Miller TS, Brett DJL (2021). Engineering catalyst layers for next generation polymer electrolyte fuel cells: A review of design, materials, and methods. Adv. Energy Mater..

[17] Randall CR, DeCaluwe SC (2020). Physically based modeling of PEMFC cathode catalyst layers: Effective microstructure and ionomer structure-property relationship impacts. J. Electrochem. Energy Convers. Storage.

[18] Kusoglu A, Weber AZ (2017). New insights into perfluorinated sulfonic-acid ionomers. Chem. Rev..

[19] Lopez-Haro M, Guétaz L, Printemps T, Morin A, Escribano S, Jouneau P-H, Bayle-Guillemaud P, Chandezon F, Gebel G (2014). Three-dimensional analysis of Nafion layers in fuel cell electrodes. Nat. Commun..

[20] Morawietz T, Handl M, Oldani C, Friedrich KA, Hiesgen R (2016). Quantitative in situ analysis of ionomer structure in fuel cell catalytic layers. ACS Appl. Mater. Interfaces.

[21] Komini Babu S, Chung HT, Zelenay P, Litster S (2016). Resolving electrode morphology’s impact on platinum group metal-free cathode performance using nano-CT of 3d hierarchical pore and ionomer distribution. ACS Appl. Mater. Interfaces.

[22] Sun C-N, More KL, Zawodzinski TA (2010). Investigation of transport properties, microstructure, and thermal behavior of PEFC catalyst layers. ECS Trans..

[23] Park Y-C, Tokiwa H, Kakinuma K, Watanabe M, Uchida M (2016). Effects of carbon supports on Pt distribution, ionomer coverage and cathode performance for polymer electrolyte fuel cells. J. Power Sources.

[24] Doo G, Lee JH, Yuk S, Choi S, Lee D-H, Lee DW, Kim HG, Kwon SH, Lee SG, Kim H-T (2018). Tuning the ionomer distribution in the fuel cell catalyst layer with scaling the ionomer aggregate size in dispersion. ACS Appl. Mater. Interfaces.

[25] Susac D, Berejnov V, Hitchcock AP, Stumper J (2011). STXM study of the ionomer distribution in the PEM fuel cell catalyst layers. ECS Trans..

[26] Zeng R, Zhang HY, Liang SZ, Wang LG, Jiang LJ, Liu XP (2020). Possible scenario of forming a catalyst layer for proton exchange membrane fuel cells. RSC Adv..

[27] Xie Z, Zhao X, Adachi M, Shi Z, Mashio T, Ohma A, Shinohara K, Holdcroft S, Navessin T (2008). Fuel cell cathode catalyst layers from “green” catalyst inks. Energy Environ. Sci..

[28] Xie J, Xu F, Wood DL, More KL, Zawodzinski TA, Smith WH (2010). Influence of ionomer content on the structure and performance of PEFC membrane electrode assemblies. Electrochim. Acta.

[29] Lee M, Uchida M, Yano H, Tryk DA, Uchida H, Watanabe M (2010). New evaluation method for the effectiveness of platinum/carbon electrocatalysts under operating conditions. Electrochim. Acta.

[30] Holdcroft S (2014). Fuel cell catalyst layers: A polymer science perspective. Chem. Mater..

[31] Berlinger SA, Garg S, Weber AZ (2021). Multicomponent, multiphase interactions in fuel-cell inks. Curr. Opin. Electrochem..

[32] Sharma R, Andersen SM (2018). Zoom in catalyst/ionomer interface in polymer electrolyte membrane fuel cell electrodes: Impact of catalyst/ionomer dispersion media/solvent. ACS Appl. Mater. Interfaces.

[33] Van Cleve T, Khandavalli S, Chowdhury A, Medina S, Pylypenko S, Wang M, More KL, Kariuki N, Myers DJ, Weber AZ, Mauger SA, Ulsh M, Neyerlin KC (2019). Dictating Pt-based electrocatalyst performance in polymer electrolyte fuel cells, from formulation to application. ACS Appl. Mater. Interfaces.

[34] Orfanidi A, Rheinländer PJ, Schulte N, Gasteiger HA (2018). Ink solvent dependence of the ionomer distribution in the catalyst layer of a PEMFC. J. Electrochem. Soc..

[35] Ott S, Orfanidi A, Schmies H, Anke B, Nong HN, Hübner J, Gernert U, Gliech M, Lerch M, Strasser P (2020). Ionomer distribution control in porous carbon-supported catalyst layers for high-power and low Pt-loaded proton exchange membrane fuel cells. Nat. Mater..

[36] Weber AZ, Borup RL, Darling RM, Das PK, Dursch TJ, Gu W, Harvey D, Kusoglu A, Litster S, Mench MM, Mukundan R, Owejan JP, Pharoah JG, Secanell M, Zenyuk IV (2014). A critical review of modeling transport phenomena in polymer-electrolyte fuel cells. J. Electrochem. Soc..

[37] Bruggeman DAG (1935). Berechnung verschiedener physikalischer Konstanten von heterogenen Substanzen. I. Dielektrizitätskonstanten und Leitfähigkeiten der Mischkörper aus isotropen Substanzen. Annalen der Physik.

[38] Tjaden B, Cooper SJ, Brett DJ, Kramer D, Shearing PR (2016). On the origin and application of the Bruggeman correlation for analysing transport phenomena in electrochemical systems. Curr. Opin. Chem. Eng..

[39] Davis HT (1977). The effective medium theory of diffusion in composite media. J. Am. Ceram. Soc..

[40] Cernuschi F, Ahmaniemi S, Vuoristo P, Mäntylä T (2004). Modelling of thermal conductivity of porous materials: Application to thick thermal barrier coatings. J. Eur. Ceram. Soc..

[41] Chalapat K, Timonen JVI, Huuppola M, Koponen L, Johans C, Ras RHA, Ikkala O, Oksanen MA, Seppälä E, Paraoanu GS (2014). Ferromagnetic resonance in ε-Co magnetic composites. Nanotechnology.

[42] Pant LM, Gerhardt MR, Macauley N, Mukundan R, Borup RL, Weber AZ (2019). Along-the-channel modeling and analysis of PEFCs at low stoichiometry: Development of a 1+2d model. Electrochim. Acta.

[43] Gloaguen F, Durand R (1997). Simulations of PEFC cathodes: An effectiveness factor approach. J. Appl. Electrochem..

[44] Boyer C, Gamburzev S, Velev O, Srinivasan S, Appleby A (1998). Measurements of proton conductivity in the active layer of PEM fuel cell gas diffusion electrodes. Electrochim. Acta.

[45] Sánchez-Ramos A, Gostick JT, García-Salaberri PA (2021). Modeling the effect of low Pt loading cathode catalyst layer in polymer electrolyte fuel cells: Part I. Model formulation and validation. J. Electrochem. Soc..

[46] Weisbrod KR (1995). Through-the-electrode model of a proton exchange membrane fuel cell. ECS Proc. Vol..

[47] Hashin Z, Shtrikman S (1962). A variational approach to the theory of the effective magnetic permeability of multiphase materials. J. Appl. Phys..

[48] Das PK, Li X, Liu Z-S (2010). Effective transport coefficients in PEM fuel cell catalyst and gas diffusion layers: Beyond Bruggeman approximation. Appl. Energy.

[49] Broadbent SR, Hammersley JM (1957). Percolation processes: I. Crystals and mazes. Math. Proc. Camb. Philos. Soc..

[50] Hunt, A. G., Ewing, R. P., & Ghanbarian, B. Percolation theory for flow in porous media. No. volume 880 in Lecture notes in physics, 3rd ed. (Springer, 2014). OCLC: ocn871300236.

[51] Stauffer D, Aharony A (2018). Introduction to Percolation Theory.

[52] Liu Y, Jorne J, Gu W (2010). Thin shell model for proton conduction in PEM fuel cell cathodes. J. Electrochem. Soc..

[53] Joshi MY (1974). A Class of Stochastic Models for Porous Media.

[54] Quiblier JA (1984). A new three-dimensional modeling technique for studying porous media. J. Colloid Interface Sci..

[55] Sabharwal M, Pant LM, Putz A, Susac D, Jankovic J, Secanell M (2016). Analysis of catalyst layer microstructures: From imaging to performance. Fuel Cells.

[56] Khakbazbaboli, M. Development of a Micro-scale Cathode Catalyst Layer Model of Polymer Electrolyte Membrane Fuel Cell. PhD thesis, Queen’s University, Kingston, ON, Canada, (2013).

[57] Barreiros Salvado M, Schott P, Guétaz L, Gerard M, David T, Bultel Y (2021). Towards the understanding of transport limitations in a proton-exchange membrane fuel cell catalyst layer: Performing agglomerate scale direct numerical simulations on electron-microscopy-based geometries. J. Power Sources.

[58] Zheng W, Kim SH (2018). The effects of catalyst layer microstructure and water saturation on the effective diffusivity in PEMFC. J. Electrochem. Soc..

[59] Mukherjee PP, Wang C-Y, Kang Q (2009). Mesoscopic modeling of two-phase behavior and flooding phenomena in polymer electrolyte fuel cells. Electrochim. Acta.

[60] Sui, P.-C., Chen, L.-D., Seaba, J. P., & Wariishi, Y. Modeling and optimization of a PEMFC catalyst layer. *SAE Trans.* 729–737, (1999).

[61] Hattori T, Suzuki A, Sahnoun R, Koyama M, Tsuboi H, Hatakeyama N, Endou A, Takaba H, Kubo M, Del Carpio CA, Miyamoto A (2008). Development of the overpotential simulator for polymer electrolyte fuel cells and application for optimization of cathode structure. Appl. Surf. Sci..

[62] Kim SH, Pitsch H (2009). Reconstruction and effective transport properties of the catalyst layer in PEM fuel cells. J. Electrochem. Soc..

[63] Siddique N, Liu F (2010). Process based reconstruction and simulation of a three-dimensional fuel cell catalyst layer. Electrochim. Acta.

[64] Lange KJ, Sui P-C, Djilali N (2010). Pore scale simulation of transport and electrochemical reactions in reconstructed PEMFC catalyst layers. J. Electrochem. Soc..

[65] Lange KJ, Sui P-C, Djilali N (2012). Determination of effective transport properties in a PEMFC catalyst layer using different reconstruction algorithms. J. Power Sources.

[66] Inoue G, Ohnishi T, So M, Park K, Ono M, Tsuge Y (2019). Simulation of carbon black aggregate and evaluation of ionomer structure on carbon in catalyst layer of polymer electrolyte fuel cell. J. Power Sources.

[67] Lange KJ, Carlsson H, Stewart I, Sui P-C, Herring R, Djilali N (2012). PEM fuel cell CL characterization using a standalone FIB and SEM: Experiments and simulation. Electrochim. Acta.

[68] Epting WK, Gelb J, Litster S (2012). Resolving the three-dimensional microstructure of polymer electrolyte fuel cell electrodes using nanometer-scale X-ray computed tomography. Adv. Funct. Mater..

[69] Thiele S, Fürstenhaupt T, Banham D, Hutzenlaub T, Birss V, Ziegler C, Zengerle R (2013). Multiscale tomography of nanoporous carbon-supported noble metal catalyst layers. J. Power Sources.

[70] Zhang X, Ostadi H, Jiang K, Chen R (2014). Reliability of the spherical agglomerate models for catalyst layer in polymer electrolyte membrane fuel cells. Electrochim. Acta.

[71] Goswami N, Mistry AN, Grunewald JB, Fuller TF, Mukherjee PP (2020). Corrosion-induced microstructural variability affects transport-kinetics interaction in PEM fuel cell catalyst layers. J. Electrochem. Soc..

[72] Iden H, Ohma A (2013). An in situ technique for analyzing ionomer coverage in catalyst layers. J. Electroanal. Chem..

[73] Takeshita T, Kamitaka Y, Shinozaki K, Kodama K, Morimoto Y (2020). Evaluation of ionomer coverage on Pt catalysts in polymer electrolyte membrane fuel cells by CO stripping voltammetry and its effect on oxygen reduction reaction activity. J. Electroanal. Chem..

[74] Eikerling M (2006). Water management in cathode catalyst layers of PEM fuel cells. J. Electrochem. Soc..

[75] Mashio T, Sato K, Ohma A (2014). Analysis of water adsorption and condensation in catalyst layers for polymer electrolyte fuel cells. Electrochim. Acta.

[76] Soboleva T, Zhao X, Malek K, Xie Z, Navessin T, Holdcroft S (2010). On the micro-, meso-, and macroporous structures of polymer electrolyte membrane fuel cell catalyst layers. ACS Appl. Mater. Interfaces.

[77] Gostick J, Khan Z, Tranter T, Kok M, Agnaou M, Sadeghi M, Jervis R (2019). PoreSpy: A python toolkit for quantitative analysis of porous media images. J. Open Source Softw..

[78] Olbrich W, Kadyk T, Sauter U, Eikerling M (2022). Modeling of wetting phenomena in cathode catalyst layers for PEM fuel cells. Electrochim. Acta.

[79] Modestov AD, Kapustin AV, Avakov VB, Landgraf IK, Tarasevich MR (2014). Cathode catalyst layers with ionomer to carbon mass ratios in the range 0–2 studied by electrochemical impedance spectroscopy, cyclic voltammetry, and performance measurements. J. Power Sources.

[80] Epting WK, Litster S (2012). Effects of an agglomerate size distribution on the PEFC agglomerate model. Int. J. Hydrogen Energy.

[81] Jankovic J, Zhang S, Putz A, Saha MS, Susac D (2019). Multiscale imaging and transport modeling for fuel cell electrodes. J. Mater. Res..

[82] Sadeghi, M. A. *et al.* Predicting pemfc performance from a volumetric image of catalyst layer structure using pore network modeling, (Under review), (2023).

[83] Virtanen P, Gommers R, Oliphant TE, Haberland M, Reddy T, Cournapeau D, Burovski E, Peterson P, Weckesser W, Bright J, van der Walt SJ, Brett M, Wilson J, Millman KJ, Mayorov N, Nelson ARJ, Jones E, Kern R, Larson E, Carey CJ, Polat I, Feng Y, Moore EW, VanderPlas J, Laxalde D, Perktold J, Cimrman R, Henriksen I, Quintero EA, Harris CR, Archibald AM, Ribeiro AH, Pedregosa F, van Mulbregt P, SciPy 1.0 Contributors (2020). SciPy 1.0: Fundamental algorithms for scientific computing in python. Nat. Methods.

[84] Cooper S, Bertei A, Shearing P, Kilner J, Brandon N (2016). TauFactor: An open-source application for calculating tortuosity factors from tomographic data. SoftwareX.

[85] Qi Y, Liu J, Sabarirajan DC, Huang Y, Perego A, Haug AT, Zenyuk IV (2021). Interpreting ionic conductivity for polymer electrolyte fuel cell catalyst layers with electrochemical impedance spectroscopy and transmission line modeling. J. Electrochem. Soc..

[86] Suzuki T, Murata H, Hatanaka T, Morimoto Y (2003). Analysis of the catalyst layer of polymer electrolyte fuel cells. R &D Rev. Toyota CRDL.

[87] Du C, Shi P, Cheng X, Yin G (2004). Effective protonic and electronic conductivity of the catalyst layers in proton exchange membrane fuel cells. Electrochem. Commun..

[88] Havránek A, Wippermann K (2004). Determination of proton conductivity in anode catalyst layers of the direct methanol fuel cell (DMFC). J. Electroanal. Chem..

[89] Paul DK, Fraser A, Karan K (2011). Towards the understanding of proton conduction mechanism in PEMFC catalyst layer: Conductivity of adsorbed Nafion films. Electrochem. Commun..

[90] Siroma Z, Kakitsubo R, Fujiwara N, Ioroi T, Yamazaki S-I, Yasuda K (2009). Depression of proton conductivity in recast Nafion®film measured on flat substrate. J. Power Sources.

[91] Karan K (2019). Interesting facets of surface, interfacial, and bulk characteristics of perfluorinated ionomer films. Langmuir.

[92] Shrivastava UN, Fritzsche H, Karan K (2018). Interfacial and bulk water in ultrathin films of Nafion, 3m PFSA, and 3m PFIA ionomers on a polycrystalline platinum surface. Macromolecules.

[93] Nagao Y (2017). Proton-conductivity enhancement in polymer thin films. Langmuir.

[94] Ono Y, Nagao Y (2016). Interfacial structure and proton conductivity of Nafion at the Pt-deposited surface. Langmuir.

[95] Andersen SM, Grahl-Madsen L (2016). Interface contribution to the electrode performance of proton exchange membrane fuel cells—Impact of the ionomer. Int. J. Hydrogen Energy.

[96] Orfanidi A, Madkikar P, El-Sayed HA, Harzer GS, Kratky T, Gasteiger HA (2017). The key to high performance low Pt loaded electrodes. J. Electrochem. Soc..

