# Supplementary material for: Structure and conductivity of ionomer in PEM fuel cell catalyst layers: a model-based analysis
Source: Sci Rep. 2023 Aug 29;13:14127. doi: 10.1038/s41598-023-40637-0 (PMC10465542; doi:10.1038/s41598-023-40637-0)
Supplement: Supplementary file 1 — Supplementary Figures. [file 41598_2023_40637_MOESM1_ESM.pdf]

Supplementary Material

The results from the convergence tests of the simulation in Fig. S1 indicate that for an image size of $L \geq 200\,nm$ with a resolution of $2\,nm$ edge length per cubic volume element results are reproducible and stable over various simulation instances. The same limitations were found before by Lange et al. (see Ref. 62, 63, and 65 in the manuscript). Lowering the resolution to only capture details above $3\,nm$ results in disconnection of voxels at curved geometries, i.e., the connectedness of the ionomer film covering the agglomerates is not well captured anymore, resulting in an underestimation of proton conductivity. Further, please note that due to the cubic complexity of the simulation the computation time excessively grows with $\mathcal{O}\left(\left(\frac{L}{vx.resol.}\right)^3\right)$. Therefore, a further increase of the simulation domain size or resolution only slightly improves simulation results, but causes significantly higher computational costs.

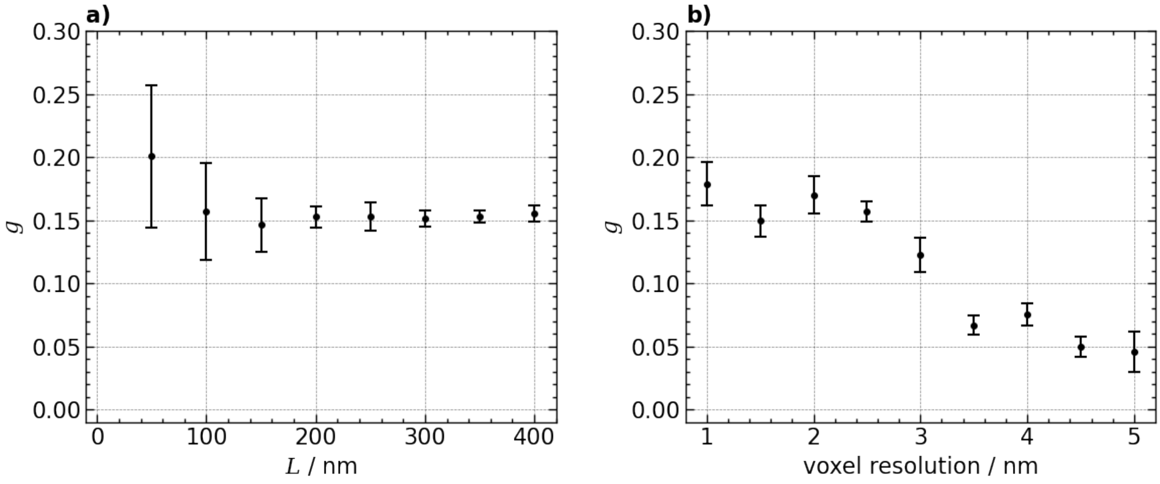

Figure **S1**: Simulation results and standard deviation from 10 samples for **a)** variation of the simulated image size at constant voxel resolution of $2\,nm$, and **b)** the voxel resolution of the image ($L = const. = 200\,nm$).

The results from sensitivity tests regarding the complete set of structural parameters used to generate the CL images are plotted in Figure S2. As discussed by Lange et al., the agglomerate surface roughness can alter the ionomer film conductivity, as it introduces additional tortuosity. However, the sensitivity for that source or error was not found to be severe for most points in the parameter space, having an impact within the statistical error margin of the simulation for carbon particle radius, ionomer patch radius, protrusion and overlap of the carbon particles. It was found that the agglomerate size (Fig S2b) must not be smaller than the carbon particle size, which would lead to unphysical simulation results, as the carbon particles are the building blocks of agglomerates. The algorithm overrides this mismatch by placing a carbon particle anyway, thus introducing more surface area and carbon volume than allowed, thereby sharply raising proton conductivity. At reduced coverage, the size of ionomer 'patches' (Fig. S2c) seems to be a sensible parameter. As percolation phenomena occur, this might be a finite size effect. Further, the proton conductivity exhibits a slightly negative trend for threshold for primary pores (Fig. S2d), which can be explained the closing of more secondary pore space and reducing the surface area of agglomerates, thus, depositing less ionomer contributing proton conductivity.

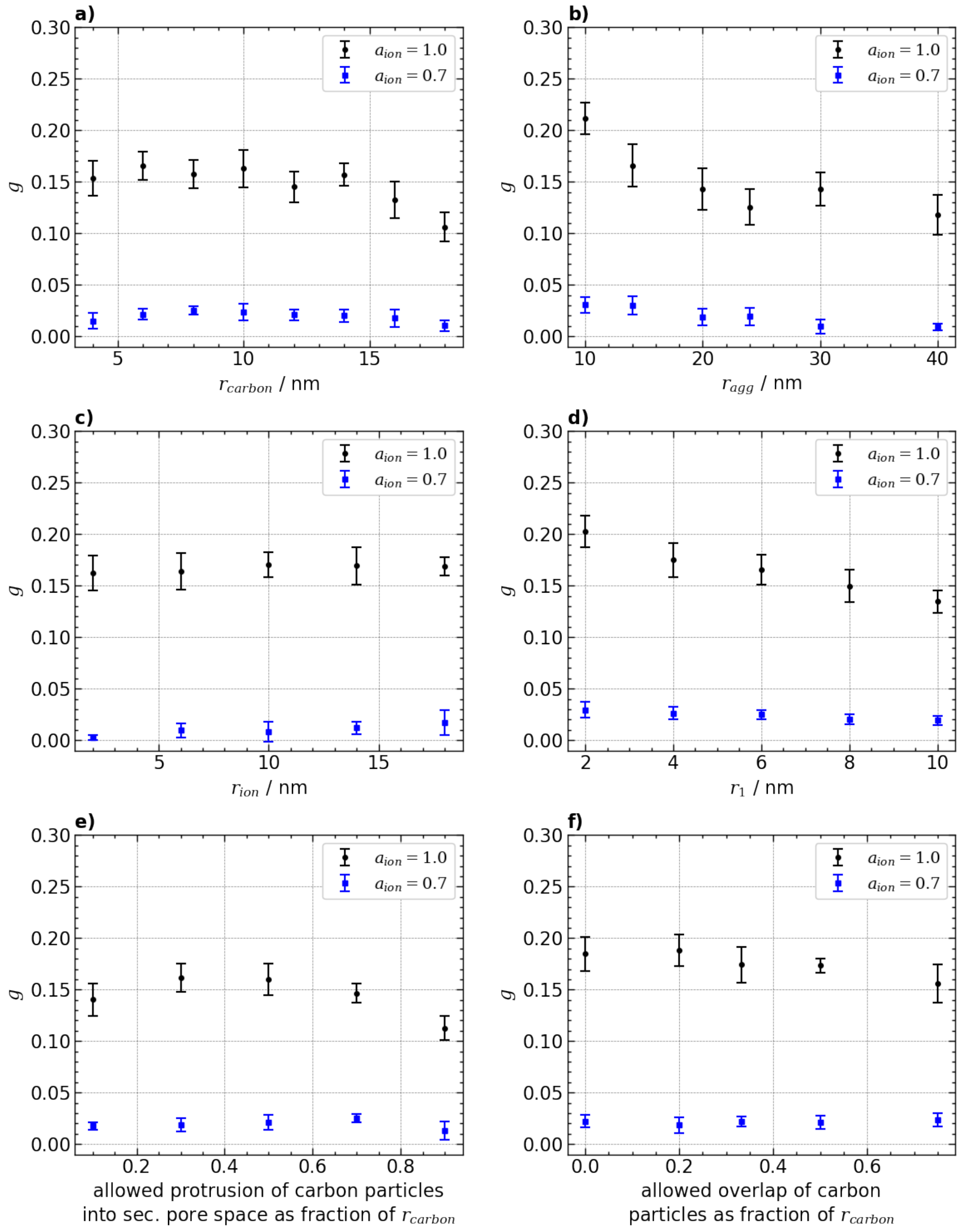

Figure **S2**: Variation of parameters from image generation to test for their sensitivity at both partial and full ionomer coverage $a_{ion} = \{0.7, 1.0\}$, including **a)** carbon particle radius, **b)** agglomerate size, **c)** ionomer patch size, **d)** primary pores radius threshold, **e)** allowed protrusion of carbon particles into secondary pore space, and **f)** allowed overlap of carbon particles. Error margins indicate standard deviation for 10 instances of the simulation.
